# Supplementary material for: Global impact of tobacco control policies on smokeless tobacco use: a systematic review protocol
Source: BMJ Open. 2020 Dec 24;10(12):e042860. doi: 10.1136/bmjopen-2020-042860 (PMC7768955; doi:10.1136/bmjopen-2020-042860)
Supplement: Supplementary data [file bmjopen-2020-042860supp001.pdf]

# SUPPLEMENTARY FILE 1_SCIENTIFIC LITERATURE SEARCH STRATEGY

Database: Ovid MEDLINE(R) ALL <1946 to April 14, 2020>

Search Strategy:

--------------------------------------------------------------------------------

1 Tobacco, Smokeless/ (3622)

2 (tobacco adj4 (SLT or ST)).ti,ab,kw. (543)

3 (tobacco adj4 (chew$ or dipping or eating or loose-leaf or oral or plug or sneeze or tablet$ or toothpaste or twist$)).ti,ab,kw. (2647)

4 (al-shammah or chemma or chimo or el-shama or ghutkha or gudakhu or gul or gutkha or gutka or iqmik or jarda or khaini or khiwam or kiwan or makla or mawa or mishri or nass or nasvay or naswar or neffa or nufha or paan or quid or sadapata or shammah or snuff or snuif or snus or taaba or tambakoo or tenfeha or tombak or tombol or toombak or tuibur or vizapatta or zarda).ti,bt,ab,kw. (4355)

5 (pan adj4 (tobacco or smokeless)).ti,ab,kw. (75)

6 (nas adj4 (tobacco or smokeless)).ti,ab,kw. (5)

7 1 or 2 or 3 or 4 or 5 or 6 (8157)

8 Public Policy/ or Government Regulation/ or Social Control, Formal/ or Taxation/ (62112)

9 (policies or policy or regulation$ or rule$ or law$ or legislation or tax or taxation or taxes).ti,ab. (1379493)

10 (strateg$ or program$).ti,ab. (1835226)

11 (ban or bans or banning or banned).ti,ab. (14063)

12 Advertising/ or Commerce/ or Marketing/ or Product Packaging/ (44071)

13 (price or prices or pricing or packaging or advertis$ or marketing or sponsor$).ti,ab. (118491)

14 Health Education/ or Health Promotion/ or Mass Media/ (134812)

15 (health warning$ or campaign$ or labelling or label or labels).ti,ab. (213540)

16 (product adj2 regulat$).ti,ab. (1080)

17 (content adj2 disclos$).ti,ab. (65)

18 illicit trade.ti,ab. (118)

19 ((sale$ or selling or sold) adj3 (minor$ or child$)).ti,ab. (390)

20 Smoking Cessation/ (28128)

21 8 or 9 or 10 or 11 or 12 or 13 or 14 or 15 or 16 or 17 or 18 or 19 or 20 (3456116)

22 7 and 21 (2199)



Arora M, *et al. BMJ Open* 2020; 10:e042860. doi: 10.1136/bmjopen-2020-042860

23 (smokeless tobacco adj3 (cessation or prevent$ or stop$)).ti,ab. (127)

24 22 or 23 (2256)

25 neonatal abstinence syndrome.ti,ab. (916)

26 NASS Patient Satisfaction Index.ti,ab. (5)

27 Normal Astacus saline.ti,ab. (1)

28 naphthenic acid.ti,ab. (136)

29 nicotianamine synthase.ti,ab. (82)

30 25 or 26 or 27 or 28 or 29 (1140)

31 24 not 30 (2253)

32 limit 31 to yr="2005 -Current" (1601)

***************************

<1>

UI - 32279158

TI - Baseline characteristics of American Indian smokeless tobacco users participating in two pilot cessation studies.

SO - Journal of Community Health. 2020 Apr 11

AU - Lewis CS

AU - Nazir N

AU - Daley SM

AU - Pacheco J

AU - Goeckner RT

AU - Hale JW

AU - Gunville JA

AU - Rahman F

AU - Choi WS

AU - Daley CM

FA - Lewis, Charley S

FA - Nazir, Niaman

FA - Daley, Sean M

FA  - Pacheco, Joseph

FA  - Goeckner, Ryan T

FA  - Hale, Jason W

FA  - Gunville, Jordyn A

FA  - Rahman, Fatima

FA  - Choi, Won S

FA  - Daley, Christine Makosky

PT  - Journal Article
