# Supplementary material for: Global impact of tobacco control policies on smokeless tobacco use: a systematic review protocol
Source: BMJ Open. 2020 Dec 24;10(12):e042860. doi: 10.1136/bmjopen-2020-042860 (PMC7768955; doi:10.1136/bmjopen-2020-042860)
Supplement: Supplementary data [file bmjopen-2020-042860supp002.pdf]

**PRISMA-P (Preferred Reporting Items for Systematic review and Meta-Analysis Protocols) 2015 checklist: recommended items to address in a systematic review protocol***

| Section and topic | Item No | Checklist item | Status | Corresponding Page number/Line number in the Manuscript |
|---|---|---|---|---|
| **ADMINISTRATIVE INFORMATION** | | | | |
| Title: | | | | |
| Identification | 1a | Identify the report as a protocol of a systematic review | ✓ | Page 3, Line 3 |
| Update | 1b | If the protocol is for an update of a previous systematic review, identify as such | Not Applicable | |
| Registration | 2 | Mention Registration Number (such as for PROSPERO) | CRD42020191946 | Page 2, Line 27 |
| Authors: | | | | |
| Contact | 3a | Provide name, institutional affiliation, e-mail address of all protocol authors; provide physical mailing address of corresponding author | ✓ | Page 1, Line 4-21 |
| Contributions | 3b | Describe contributions of protocol authors and identify the guarantor of the review | ✓ | |
| Amendments | 4 | If the protocol represents an amendment of a previously completed or published protocol, identify as such and list changes; otherwise, state plan for documenting important protocol amendments | Not Applicable | Page 14, Line 2-4 |
| Support: | | | | |
| Sources | 5a | Indicate sources of financial or other support for the review | ✓ | Page 14, Line 5-7 |
| Sponsor | 5b | Provide name for the review funder and/or sponsor | ✓ | Page 14, Line 5-7 |
| Role of sponsor or funder | 5c | Describe roles of funder(s), sponsor(s), and/or institution(s), if any, in developing the protocol | ✓ | Page 1, Line 16-21; and Page 14, Line 5-7 |
| **INTRODUCTION** | | | | |
| Rationale | 6 | Describe the rationale for the review in the context of what is already known | ✓ | Page 5, Line 11-14 |
| Objectives | 7 | Provide an explicit statement of the question(s) the review will address with reference to participants, interventions, comparators, and outcomes (PICO) | ✓ | Page 5, Line 16-24 |



Arora M, *et al. BMJ Open* 2020; 10:e042860. doi: 10.1136/bmjopen-2020-042860

| | | | | |
|---|---|---|---|---|
| **METHODS** | | | | |
| Eligibility criteria | 8 | Specify the study characteristics (such as PICO, study design, setting, time frame) and report characteristics (such as years considered, language, publication status) to be used as criteria for eligibility for the review | ✓ | Page 5, Line 26-34; Page 6 Line 1-32; Page 7, Line 1-33; Page 8, Line 1-16; Page 7, Line 25-27 |
| Information sources | 9 | Describe all intended information sources (such as electronic databases, contact with study authors, trial registers or other grey literature sources) with planned dates of coverage | ✓ | Page 7, Line 29-33; Page 8, Line 1-16 |
| Search strategy | 10 | Present draft of search strategy to be used for at least one electronic database, including planned limits, such that it could be repeated | ✓ | Page 8, Line 18-34; Page 9, Line 1-25 |
| Study records: | | | | |
| Data management | 11a | Describe the mechanism(s) that will be used to manage records and data throughout the review | ✓ | Page 9, Line 27-29 |
| Selection process | 11b | State the process that will be used for selecting studies (such as two independent reviewers) through each phase of the review (that is, screening, eligibility and inclusion in meta-analysis) | ✓ | Page 9, Line 31-34 |
| Data collection process | 11c | Describe planned method of extracting data from reports (such as piloting forms, done independently, in duplicate), any processes for obtaining and confirming data from investigators | ✓ | Page 10, Line 1-8 |
| Data items | 12 | List and define all variables for which data will be sought (such as PICO items, funding sources), any pre-planned data assumptions and simplifications | ✓ | Page 10, Line 18-32 |
| Outcomes and prioritization | 13 | List and define all outcomes for which data will be sought, including prioritization of main and additional outcomes, with rationale | ✓ | Page 7, Line 7-23 |
| Risk of bias in individual studies | 14 | Describe anticipated methods for assessing risk of bias of individual studies, including whether this will be done at the outcome or study level, or both; state how this information will be used in data synthesis | ✓ | Page 10, Line 10-15 |
| Data synthesis | 15a | Describe criteria under which study data will be quantitatively synthesised | ✓ | Page 10, Line 17-32; Page 11 Line 1-16 |
| | 15b | If data are appropriate for quantitative synthesis, describe planned summary measures, methods of handling data and methods of combining data from studies, including any planned exploration | ✓ | |

| | | | | |
|---|---|---|---|---|
| | | of consistency (such as $I^2$, Kendall's $\tau$) | | |
| | 15c | Describe any proposed additional analyses (such as sensitivity or subgroup analyses, meta-regression) | | Page 11, Line 1-16 |
| | 15d | If quantitative synthesis is not appropriate, describe the type of summary planned | ✓ (described for narrative synthesis) | Page 10, Line 18-27 |
| Meta-bias(es) | 16 | Specify any planned assessment of meta-bias(es) (such as publication bias across studies, selective reporting within studies) | ✓ | Page 11, Line 1-8 |
| Confidence in cumulative evidence | 17 | Describe how the strength of the body of evidence will be assessed (such as GRADE) | ✓ | Page 10, Line 17-32; Page 11 Line 1-16 |

*** It is strongly recommended that this checklist be read in conjunction with the PRISMA-P Explanation and Elaboration (cite when available) for important clarification on the items. Amendments to a review protocol should be tracked and dated. The copyright for PRISMA-P (including checklist) is held by the PRISMA-P Group and is distributed under a Creative Commons Attribution Licence 4.0.**

*From: Shamseer L, Moher D, Clarke M, Ghersi D, Liberati A, Petticrew M, Shekelle P, Stewart L, PRISMA-P Group. Preferred reporting items for systematic review and meta-analysis protocols (PRISMA-P) 2015: elaboration and explanation. BMJ. 2015 Jan 2;349(jan02 1):g7647.*
