# Supplementary material for: Global impact of tobacco control policies on smokeless tobacco use: a systematic review protocol
Source: BMJ Open. 2020 Dec 24;10(12):e042860. doi: 10.1136/bmjopen-2020-042860 (PMC7768955; doi:10.1136/bmjopen-2020-042860)
Supplement: Supplementary data [file bmjopen-2020-042860supp003.pdf]

Addressing Smokeless Tobacco and Building Capacity in South Asia (ASTRA) – Policy Workstream

Data Extraction Form

**SECTION I: General Information and Identification**

1.	Title of the Article/ Document	
2.	Study ID *(surname of first author and year study was published e.g. Smith 2001)*	
3.	Report ID *(for projects or studies with multiple report - if different to Study ID e.g. Smith 2001_01)*	
4.	Report IDs of other reports of this study *(e.g. duplicate publications, follow-up studies)*	
5.	Date form completed (dd/mm/yyyy)	
6.	Initials of person extracting data	
7.	Full reference with URL	
8.	Type of Document	☐Scientific article<br>☐Government Report<br>☐Policy Document<br>☐Non-government report<br>☐Commentary<br>☐Editorial<br>☐Government Circular<br>☐Others
9.	Study author contact details	
10.	Source of document<br>*(If ministry website, mention which ministry – Health, Environment, Commerce etc.)*	☐Academic journal<br>☐Ministry website, name: ____________________

		☐Google<br>☐Other, name:____<br>______________
11.	Country (in which study was conducted/policy document is based)	
12.	Duration of study *(start and end date)*	
13.	State funding source	
14.	Ethics approval obtained for the study (Y/N)	

SECTION II: DETAILS OF SMOKELESS TOBACCO POLICY (specific to RQ1)

1.	Population (study participants)	☐Smokers<br>☐Smokeless Tobacco Users<br>☐Dual Users<br>☐Any other, please specify:
2.	Age group of study participants (adolescents/young adults/adults)	☐ All age groups<br>☐adults, age range: __________________________<br>☐children/youth, age range: __________________________<br>☐other, age range: __________________________<br>☐comment/warning message (if any):<br>______________________________
3.	Gender distribution of participants	Total number of males (%):<br>Total number of females (%):
4.	Setting of the population ?(national or sub-national)	
5.	Number of participants/sample size	

	**INTERVENTION (POLICY) DESCRIPTION**	
	**INTERVENTION (POLICY) 1 (replicate the entire section in case of more than 1 policy)**	
1.	Intervention (policy) focus	**FCTC Policies**<br>☐Pricing and taxation (Article 6)<br>☐Product regulation (Article 9 and 10)<br>☐Packaging and health warnings (Article 11)<br>☐Education, communication, training, and public awareness (Article 12)<br>☐Advertisement, promotion and sponsorship bans (Article 13)<br>☐Cessation (Article 14)<br>☐Illicit trade (Article 15)<br>☐Sales to and by minors (Article 16)<br>**Non-FCTC Policies**<br>☐Complete ban<br>☐Partial ban<br>☐Import ban<br>☐Other policies mentioned to control ST (agriculture, environment etc.), please specify____ ______________
2.	Comparator *(usual care/control etc.)*	☐Reported/Describe: ____<br>☐Not reported (but should be reported)<br>☐Not applicable
Description of intervention (using TIDieR checklist: https://www.equator-network.org/reporting-guidelines/tidier/)		
3.	Brief name<br>(name or phrase that describes the policy in the document)	☐Present/Describe: ____<br>☐Absent (but should be reported)

		☐Not applicable (when it is legitimately not relevant)
4.	Why?<br>(Describe any rationale, theory, or goal of the elements essential to the policy)	☐Present/Describe:<br>____<br>☐Absent (but should be reported)<br>☐Not applicable (when it is legitimately not relevant)
5.	What materials<br>(any physical or informational materials used for the policy)	☐Present/Describe:<br>____<br>☐Absent (but should be reported)<br>☐Not applicable (when it is legitimately not relevant)
6.	What procedures<br>(procedures, activities, and/or processes used in the policy)	☐Present/Describe:<br>____<br>☐Absent (but should be reported)<br>☐Not applicable (when it is legitimately not relevant)
7.	Who provided<br>(For each category of intervention provider (e.g. psychologist, nursing assistant), describe their expertise, background and any specific training given; N/A for non-human provider modes)	☐Present/Describe:<br>____<br>☐Absent (but should be reported)<br>☐Not applicable (when it is legitimately not relevant)
8.	How<br>(modes of delivery (e.g. face-to-face or by some other mechanism, such as internet or telephone) of the intervention and whether it was provided individually or in a group)	☐Present/Describe:<br>____<br>☐Absent (but should be reported)<br>☐Not applicable (when it is legitimately not relevant)
9.	Where<br>(Describe the type(s) of location(s) where the policy occurred, including any necessary infrastructure or relevant features)	☐Present/Describe:<br>____<br>☐Absent (but should be reported)<br>☐Not applicable (when it is legitimately not relevant)
10.	When and how much<br>(period of time covered by the policy and any specification on frequency and intensity)	☐Present/Describe:<br>____<br>☐Absent (but should be reported)<br>☐Not applicable (when it is legitimately not relevant)

11.	Tailoring<br><br>(If it was planned to be personalised, titrated or adapted for a specific population, then describe what, why, when, and how)	☐Present/Describe: ____________________<br>☐Absent (but should be reported)<br>☐Not applicable (when it is legitimately not relevant)
12.	Modifications<br><br>(any modified made to the policy during the course of the study, describe the changes (what, why, when, and how))	☐Present/Describe: ____________________<br>☐Absent (but should be reported)<br>☐Not applicable (when it is legitimately not relevant)
13.	How well implemented (plan)<br><br>(whether policy adherence was assessed, and if any strategies were used to maintain and improve adherence)	☐Present/Describe: ____________________<br>☐Absent (but should be reported)<br>☐Not applicable (when it is legitimately not relevant)
14.	How well implemented (actual)<br><br>(if policy adherence was assessed, describe the extent to which it was implemented as planned)	☐Present/Describe: ____________________<br>☐Absent (but should be reported)<br>☐Not applicable (when it is legitimately not relevant)
Contextual specification of intervention		
15.	Is the extent of policy enforcement described in the document?	☐Yes, National/federal level<br>☐Yes, Regional/state-level/provincial<br>☐No<br><br>If Yes, Describe the extent:________________
16.	Enforcers/regulators of the policy (Government body enforcing or regulating the policy)	☐Ministry/Department/Division of Health<br>☐Ministry/Department/Division of Commerce<br>☐Ministry/Department/Division of Finance<br>☐Ministry/Department/Division of Environment<br>☐Food and Drug Administration<br>☐ Others, please specify____ _______

17.	Does this document identify if stakeholders were involved in developing/modifying the policy?	☐Yes<br>☐No
18.	If Yes in Point 5, select all the stakeholders that were involved in developing/modifying the policy	☐Federal Government/National Government<br>☐Provincial/State/Regional Government<br>☐Health Care Organisations<br>☐Experts<br>☐Regulators<br>☐Professional Organisations (non-regulatory)<br>☐Clinicians<br>☐Patients<br>☐Researchers<br>☐Others (specify)___________________________<br>☐Not available
19.	Does the document describe any policy drivers, e.g., preamble or rationale for introducing policies (like media coverage, political will, public health concern etc.)?	☐Yes<br>☐No<br><br>if yes, specify details:
20.	Does the document evaluate or mention evaluation of the policy's effectiveness?	☐Evaluates impact *(complete Section III)*<br>☐Mentions evaluation of impact *(in methods)*<br>*State/provide reference of the article/document with details of the impact evaluation:*<br>☐None of the above
21.	Any other details (limitations or other observations)	

SECTION III: DETAILS OF IMPACT OF SMOKELESS TOBACCO POLICIES (specific to RQ2)

A. Methods

			Location in text (Page #/ Figure/Table)
1.	Study objectives *(as stated in the study)*		
2.	Design	☐Randomized controlled trial<br>☐Controlled clinical trial<br>☐Cohort analytic (two groups pre+post)<br>☐Case-control<br>Cross sectional (surveys)<br>☐Cohort (one group pre+post (before and after))<br>☐Interrupted time series<br>☐Other specify_______________________________<br>☐Not specified	
3.	Sampling technique with details	☐Random sampling<br>☐Purposive sampling<br>☐Snowball sampling<br>☐Cluster sampling<br>☐Any other, please specify:<br>______________________________<br>☐Not specified	

4.	Is the analysis of the study conducted at individual level?	☐No<br>☐Yes, please give details:_________	
5.	Is the analysis of the study conducted at group level?	☐No<br>☐Yes, please give details:___________	

B. Outcomes

			Location in text (Page #/ Figure/Table)
	**PRIMARY OUTCOME 1 (replicate the section in case of more than one PRIMARY outcome)**		
1.	Outcome name (e.g. quit rate)		
2.	Outcome definition		
3.	Time points measured		
4.	Time since policy implementation		
5.	Time points reported		

6.	Total N (% - *at this stage of follow-up as % of N at time of enrolment in study)*		
7.	N (%) with outcome		
8.	Effect estimate *(e.g. Odds Ratio/Prevalence percentage/risk ratio/mean/median)*		
9.	Unit of effect estimate *(e.g. O*dds Ratio, percentage, mean etc.*)*		
10	Confidence/precision intervals of effect estimate *(e.g. 95% CI, IQR, SD, SR etc.)*		
11	Is tool validated for population of interest		
	**INTERMEDIATE OUTCOME**		
	**Details**		
	**UNINTENDED OUTCOME**		
	**Details**		

	Notes:

C. COMPARATORS

			Location in text (Page #/ Figure/Table)
	**COMPARATOR 1 (Replicate the section in case of more than one comparator)**		
1.	Comparator Name (e.g. quit rate)		
2.	Comparator definition		
3.	Time points measured		
4.	Time since policy implementation		
5.	Time points reported		
6.	Total N (% - *at this stage of follow-up as % of N at time of enrolment in study)*		
7.	N (%) with outcome		
8.	Effect estimate *(e.g. Odds Ratio/Prevalence percentage/risk ratio/mean/median)*		

9.	Unit of effect estimate *(e.g. O*dds Ratio, percentage, mean etc.*)*		
10.	Confidence/precision intervals of effect estimate *(e.g. 95% CI, IQR, SD, SR etc.)*		

D. Limitation and Mitigation Strategy (author identified)

			Location in text (Page #/ Figure/Table)
1.	Strength		
2.	Limitation		

E. Conclusions

			Location in text (Page #/ Figure/Table)
1.	Key Conclusion of Study Author/s		

F. Risk of bias (quality assessment)

1. **SELECTION BIAS**	
a. Are the individuals selected to participate in the study likely to be representative of the target population?	☐Very likely<br>☐Somewhat likely<br>☐Not likely<br>☐Can't tell
b. What percentage of selected individuals agreed to participate?	☐80-100% agreement<br>☐60-79% agreement<br>☐Less than 60% agreement<br>☐Not applicable<br>☐Can't tell
Rate this section (selection bias)	☐1 Strong<br>☐2 Moderate<br>☐3 Weak
2. **STUDY DESIGN**	
a. Indicate the study design	☐Randomized controlled trial<br>☐Controlled clinical trial<br>☐Cohort analytic (two groups pre+post)<br>☐Case-control<br>Cross sectional (surveys)<br>☐Cohort (one group pre+post (before and after))<br>☐Interrupted time series<br>☐Other specify________________________________<br>☐Can't tell
b. Was the study described as randomized? (If No, go to component 3)	☐No<br>☐Yes

c. If yes, was the method of randomization described?	☐No<br>☐Yes
d. If yes, was the method appropriate?	☐No<br>☐Yes
Rate this section (study design)	☐1 Strong<br>☐2 Moderate<br>☐3 Weak
3. **CONFOUNDERS**	
a. Were there important differences between groups prior to intervention?	☐Yes<br>☐No<br>☐Can't tell
The following are examples of confounders	☐Race<br>☐Sex<br>☐Marital status/family<br>☐Age<br>☐SES (income or class)<br>☐Education<br>☐Health status<br>☐Pre-intervention score on outcome measure
b. If yes, indicate the percentage of relevant confounders that were controlled (either in the design (e.g., stratification, matching) or analysis)?	☐80-100% (most)<br>☐60-79% (some)<br>☐Less than 60% (few or none)<br>☐Can't tell
Rate this section	☐1 Strong<br>☐2 Moderate<br>☐3 Weak
**4. BLINDING**	
a. Was (were) the outcome assessor(s) aware of the intervention or exposure status of participants?	☐Yes<br>☐No<br>☐Can't tell

b. Were the study participants aware of the research question?	☐Yes<br>☐No<br>☐Can't tell
Rate this section	☐1 Strong<br>☐2 Moderate<br>☐3 Weak
**5. DATA COLLECTION METHODS**	
a. Were data collection tools shown to be valid?	☐Yes<br>☐No<br>☐Can't tell
b. Were data collection tools shown to be reliable?	☐Yes<br>☐No<br>☐Can't tell
Rate this section	☐1 Strong<br>☐2 Moderate<br>☐3 Weak
**6. WITHDRAWALS AND DROP-OUTS**	
a. Were withdrawals and drop-outs reported in terms of numbers and/or reasons per group	☐Yes<br>☐No<br>☐Can't tell<br>☐Not applicable (i.e. one time surveys or interviews)
b. Indicate the percentage of participants completing the study (if the percentage differs by groups, record the lowest)	☐80-100%<br>☐60-79%<br>☐Less than 60%<br>☐Can't tell
Rate this section	☐1 Strong<br>☐2 Moderate<br>☐3 Weak
**7. INTERVENTION INTEGRITY**	

a. What percentage of participants received the allocated intervention or exposure of interest?	☐80-100%<br>☐60-79%<br>☐Less than 60%<br>☐Can't tell
b. Was the consistency of the intervention measured?	☐Yes<br>☐No<br>☐Can't tell
c. Is it likely that subjects received an unintended intervention (contamination or co-intervention) that may influence the results	☐Yes<br>☐No<br>☐Can't tell
**8. ANALYSES**	
a. Indicate the unit of allocation (select one)	☐Community<br>☐Organisation/institution<br>☐Practice/office<br>☐Individual
b. Indicate the unit of analysis (select one)	☐Community<br>☐Organisation/institution<br>☐Practice/office<br>☐Individual
c. Are the statistical methods appropriate for the study design?	☐Yes<br>☐No<br>☐Can't tell
d. Is the analysis performed by intervention allocation status (i.e. intention to treat) rather than the actual intervention received?	☐Yes<br>☐No<br>☐Can't tell
**COMPONENT RATINGS**	
a. Selection Bias	☐1 Strong<br>☐2 Moderate<br>☐3 Weak
b. Study Design	☐1 Strong

	☐2 Moderate<br>☐3 Weak
1 Confounders	☐1 Strong<br>☐2 Moderate<br>☐3 Weak
2 Blinding	☐1 Strong<br>☐2 Moderate<br>☐3 Weak
3 Data collection method	☐1 Strong<br>☐2 Moderate<br>☐3 Weak
4 Withdrawals and drop-outs	☐1 Strong<br>☐2 Moderate<br>☐3 Weak
**GLOBAL RATING FOR THIS PAPER (SELECT ONE)**	☐1 Strong<br>☐2 Moderate<br>☐3 Weak
(With both reviewers discussing the ratings)<br>Is there a discrepancy between the reviewers with respect to the component (a. – f.)	☐No<br>☐Yes
If yes, indicate the reason for discrepancy	☐Oversight<br>☐Differences in interpretation of criteria<br>☐Differences in interpretation of study
**Final Decision of both reviewers (select one)**	☐1 Strong<br>☐2 Moderate<br>☐3 Weak
