# Supplementary material for: Global impact of tobacco control policies on smokeless tobacco use: a systematic review protocol
Source: BMJ Open. 2020 Dec 24;10(12):e042860. doi: 10.1136/bmjopen-2020-042860 (PMC7768955; doi:10.1136/bmjopen-2020-042860)
Supplement: Author's manuscript [file bmjopen-2020-042860.draft_revisions.pdf]

BMJ Open

BMJ Open is committed to open peer review. As part of this commitment we make the peer review history of every article we publish publicly available.

When an article is published we post the peer reviewers' comments and the authors' responses online. We also post the versions of the paper that were used during peer review. These are the versions that the peer review comments apply to.

The versions of the paper that follow are the versions that were submitted during the peer review process. They are not the versions of record or the final published versions. They should not be cited or distributed as the published version of this manuscript.

BMJ Open is an open access journal and the full, final, typeset and author-corrected version of record of the manuscript is available on our site with no access controls, subscription charges or pay-per-view fees (http://bmjopen.bmj.com).

If you have any questions on BMJ Open's open peer review process please email info.bmjopen@bmj.com

BMJ Open

The global impact of tobacco control policies on smokeless tobacco use: a systematic review protocol

Journal:	*BMJ Open*
Manuscript ID	bmjopen-2020-042860
Article Type:	Protocol
Date Submitted by the Author:	16-Jul-2020
Complete List of Authors:	Arora, Monika; HRIDAY, ; Public Health Foundation of India, Health Promotion<br>Chugh, Aastha; HRIDAY<br>Jain, Neha; Public Health Foundation of India,<br>Mishu, Masuma; University of York Department of Health Sciences, Health Sciences<br>Boeckmann, Melanie; Bielefeld University, School of Public Health<br>Dahanayake, Suranji; University of York Department of Health Sciences, Health Sciences<br>Eckhardt, Jappe; University of York, Politics<br>Forberger, Sarah; Leibniz Institute for Prevention Research ,<br>Huque, Rumana; University of Dhaka, Economics<br>Kanaan, Mona; University of York, Health Sciences<br>khan, Zohaib; Khyber Medical University, ;<br>Mehrotra, Ravi; National Institute of Cancer Prevention and Research, Department of Health Research<br>Rahman, Muhammad; Federation University Australia, School of Health; La Trobe University,<br>Readshaw, Anne; University of York Department of Health Sciences, Health Sciences; University ofYork<br>Sheikh, Aziz; University of Edinburgh, Division of Community Health Sciences<br>Siddiqi, Kamran; University of York, UK, Institute of Health Sciences<br>Vidyasagaran, Aishwarya; University of York Department of Health Sciences, Health Sciences<br>Dogar, Omara; University of York, Health Sciences
Keywords:	Health policy < HEALTH SERVICES ADMINISTRATION & MANAGEMENT, Protocols & guidelines < HEALTH SERVICES ADMINISTRATION & MANAGEMENT, International health services < HEALTH SERVICES ADMINISTRATION & MANAGEMENT, Public health < INFECTIOUS DISEASES

SCHOLARONE™
Manuscripts

I, the Submitting Author has the right to grant and does grant on behalf of all authors of the Work (as defined in the below author licence), an exclusive licence and/or a non-exclusive licence for contributions from authors who are: i) UK Crown employees; ii) where BMJ has agreed a CC-BY licence shall apply, and/or iii) in accordance with the terms applicable for US Federal Government officers or employees acting as part of their official duties; on a worldwide, perpetual, irrevocable, royalty-free basis to BMJ Publishing Group Ltd ("BMJ") its licensees and where the relevant Journal is co-owned by BMJ to the co-owners of the Journal, to publish the Work in this journal and any other BMJ products and to exploit all rights, as set out in our licence.

The Submitting Author accepts and understands that any supply made under these terms is made by BMJ to the Submitting Author unless you are acting as an employee on behalf of your employer or a postgraduate student of an affiliated institution which is paying any applicable article publishing charge ("APC") for Open Access articles. Where the Submitting Author wishes to make the Work available on an Open Access basis (and intends to pay the relevant APC), the terms of reuse of such Open Access shall be governed by a Creative Commons licence – details of these licences and which Creative Commons licence will apply to this Work are set out in our licence referred to above.

Other than as permitted in any relevant BMJ Author's Self Archiving Policies, I confirm this Work has not been accepted for publication elsewhere, is not being considered for publication elsewhere and does not duplicate material already published. I confirm all authors consent to publication of this Work and authorise the granting of this licence.

For peer review only

Title: The global impact of tobacco control policies on smokeless tobacco use: a systematic review protocol

Authors: Monika Arora[1, 2], Aastha Chugh[1], Neha Jain[2], Masuma Pervin Mishu[3], Melanie Boeckmann[3,4], Suranji Dahanayake[3,5], Jappe Eckhardt[3], Sarah Forberger[6], Rumana Huque[7], Mona Kanaan[3], Zohaib Khan[8], Ravi Mehrotra[9], Muhammad Aziz Rahman[10,11], Anne Readshaw[3], Aziz Sheikh[12], Kamran Siddiqi[3], Aishwarya Vidyasagaran[3], Omara Dogar[3,12]

[1]HRIDAY (India), [2]Public Health Foundation of India (India), [3]University of York (United Kingdom),[4] Bielefeld University School of Public Health (Germany), [5] Ministry of Health, Nutrition and Indigenous Medicine (Sri Lanka), [6]Leibniz Institute For Prevention Research and Epidemiology, BIPS (Germany), [7]ARK Foundation (Bangladesh), [8]Khyber Medical University (Pakistan), [9]Indian Council of Medical Research (India),[10]Federation University Australia (Australia), [11]La Trobe University (Australia), [12]University of Edinburgh (United Kingdom)

Acknowledgements: Ian Kellar (University of Leeds, United Kingdom), Subhash Pokhrel (Brunel University, United Kingdom), ASTRA Global Health Research Group (https://www.york.ac.uk/healthsciences/research/public-health/projects/astra/)

Corresponding Author: J.-Prof. Dr. Melanie Boeckmann, Bielefeld University School of Public Health, Department of Environment and Health, 33615 Bielefeld, Germany, Email: Melanie.boeckmann@uni-bielefeld.de

Keywords: Tobacco control, Smokeless tobacco, policies, impact, systematic review

Word Count: 2965 (excluding figure and references)

For peer review only - http://bmjopen.bmj.com/site/about/guidelines.xhtml

Abstract

Introduction: Smokeless tobacco (ST) was consumed by 356 million people globally in 2017. Recent evidence shows that ST consumption is responsible for an estimated 652,494 all-cause deaths across the globe annually. The WHO FCTC was negotiated in 2003 and ratified in 2005 to implement effective tobacco control measures. While the policy measures enacted through various tobacco control laws have been effective in reducing the incidence and prevalence of smoking, the impact of ST-related policies (within WHO FCTC and beyond) on ST use is under-researched and not collated.

Methods and Analysis: A systematic review will be conducted to collate all available ST related policies implemented across various countries and assess their impact on ST use. The following databases will be searched: Medline, EMBASE , PsycINFO, Cumulative Index to Nursing and Allied Health Literature (CINAHL), Scopus, EconLit, ISI Web of Science, Cochrane Library (CENTRAL), African Index Medicus (AIM), LILACS, Scientific Electronic Library Online (SciELO), Index Medicus for the Eastern Mediterranean Region (IMEMR), Index Medicus for South-East Asia Region (IMSEAR), Western Pacific Region Index Medicus (WPRIM), and WHO Library Database (WHOLIS); as well as Google search engine and country-specific government websites. All ST related policy documents (FCTC and non-FCTC) will be included. Results will be limited to literature published since 2005 in English and regional languages (Bengali, Hindi and Urdu). Two reviewers will independently employ two-stage screening to determine inclusion. The Effective Public Health Practice Project (EPHPP)'s "Quality Assessment Tool for Quantitative Studies" will be used to record ratings of quality and risk of bias among studies selected for inclusion. Data will be extracted using a standardised form. Meta-analysis and narrative synthesis will be used.

Ethics: Permission for ethics exemption of the review was obtained from the Centre for Chronic Disease Control's Institutional Ethics Committee, India (CCDC_IEC_06_2020; dated 16 April 2020).

PROSPERO Registration Number: CRD42020191946

Article Summary

Strengths and limitations of the study:

- The study is a comprehensive global review of all available ST related policies.

For peer review only - http://bmjopen.bmj.com/site/about/guidelines.xhtml

- The review will include all available ST related policies (FCTC and non-FCTC policies). It will also include ST policies notified/enforced by ministries other than health such as Ministry of Finance and Ministry of Commerce.
- This review is the first attempt to search for ST related policies globally and assess their impact systematically across academic databases and country-specific government websites.
- Studies and policy documents in English and regional languages for which expertise is available in the team (Bangla, Hind, and Urdu) will be included in the review. Due to limited resources, documents in other languages will not be included.

For peer review only

Introduction

Tobacco products are broadly classified into two categories – smoked and smokeless tobacco (ST). The World Health Organization's Framework Convention on Tobacco Control (WHO FCTC) defines ST as "tobacco that is consumed in un-burnt form either orally or nasally".[1] A wide range of ST products are manufactured and consumed

For peer review only - http://bmjopen.bmj.com/site/about/guidelines.xhtml

worldwide, some of which are snus, pan masala, gutkha, mawa, khaini, zarda, mishri, dry snuff, moist snuff etc. ST was consumed by 356 million people globally in 2017.[2] Cumulatively, it is estimated that, globally, a total of 652,494 people die from ST attributed diseases annually.[3] It is used in about 116 countries around the world[4], and a vast majority of users reside in South and South-East Asia, which bears more than 85% of the burden of diseases due to ST use.[5] The practice of ST use is culturally influenced and widespread in this region, where one-third of tobacco is consumed in the smokeless form.[6] This widespread use of ST products is a cause for alarm, considering that they have been known to contain more than 30 carcinogens.[6] The INTERHEART study, a large case-control study published in 2006 and undertaken in 52 countries, found that 4.7 million DALYs were lost and 204,309 people died from coronary heart diseases caused by ST consumption.[7] ST use leads to oral sub-mucous fibrosis and cancers of the oral cavity.[8] Pregnant women using ST have shown a threefold increased risk of stillbirth and two- to threefold increased risk of having low-birth-weight babies.[9–11]

The WHO FCTC was negotiated in 2003, and ratified in 2005, with the aim of providing guidelines to implement effective tobacco control measures.[1] Currently, there are 183 country-level signatories to this treaty. Though the menace of ST use is considered to be relevant to fewer countries and regions than smoking, it is evident that there has been an increase in the prevalence of ST use, even in economically-developed countries.[12] However, the situation is worse in low- and middle-income countries (LMICs), such as India, where the prevalence of ST use is higher than smoking.[13] While 10.7% of all the adults in India are smokers, 21.4% are current ST users.[13] Similarly in Bangladesh, while 18% of all adults are smokers, 20.6% are ST users.[14]

Furthermore, while the policies described under FCTC have been successful in decreasing the incidence and prevalence of smoking, there is insufficient evidence on the impact of the same on ST use.[2] For example, taxation and smoke-free policies (as described in Article 6 of the FCTC) have been successful in reducing the prevalence of smoking in countries such as Greece.[15] However, the impact of these policies on ST use is not widely known, nor have they been systematically collated. This could be attributed to the fact that ST has not been given the same importance as smoking while implementing these policies. In addition, these policies have often been based on evidence gathered from research on cigarettes conducted in high-income countries, and hence the translation of the same policies for the control of ST in LMICs has not been contextualised.[16] For

For peer review only - http://bmjopen.bmj.com/site/about/guidelines.xhtml

instance, mass media campaigns for raising awareness on ST have been highlighted by very few countries, and sale of ST products to minors is illegal only in a handful of the countries.[2,17] In addition, second hand impact of ST use (such as diseases linked to spitting) and its adverse environmental impacts due to disposal of small sachets of ST products) do not invoke any public health response. The burden of ST is now being realized in some developed countries too. For example, Australia recently imposed a ban on import, production, sale, distribution and advertisement of ST products, including chewing tobacco, oral snuff, tobacco paste and powder.[18] On other hand some countries, for example the USA, have enforced amendments to their Food and Drug Administration's (FDA's) Family Smoking Prevention and Tobacco Control Act in 2009, to consider ST a modified risk tobacco product.[12] This change in policy scenario has been neither persistent nor uniform across the world. Countries like India and Sri Lanka have gone beyond FCTC to include the prohibition of some (e.g. gutka) or all ST products.[19] The impact of FCTC policies, as well as country-specific tobacco control laws, on the consumption of ST is under-researched and not collated.

Aims and Objectives

The study aims to undertake a systematic review to collate all policies implemented across countries globally relevant to the control of ST products and assess their impact on ST use. Specific review questions include:

Review Question 1 (RQ1): What are the existing policies and legislation (FCTC and non-FCTC) related to the control of ST products across various countries globally?

Review Question 2 (RQ2): What is the impact of these policies on controlling ST use, including its uptake, quit and reduction?

Methodology

The study is a systematic review which will consider all relevant studies from 2005 onwards (after the WHO FCTC came into force) without any geographical restriction. Articles for which linguistic expertise is available within the team (Bengali, Hindi and Urdu) will be included. Our study will include published articles (including commentaries and editorials) searched through scientific databases. Policy documents (policies/laws/notifications/Acts related to ST) identified through grey literature searches will also be included in the review. Systematic reviews will be excluded because

For peer review only - http://bmjopen.bmj.com/site/about/guidelines.xhtml

we expect to cover most of the relevant literature through our searches and a review of systematic reviews will be beyond the scope of the study. Studies reporting at least one of the outcomes (detailed below) in relation to at least one policy of interest will be eligible for inclusion. We will identify studies using keywords such as ''smokeless tobacco'', ''public policies'', etc., with synonyms, and based on the PICOS criteria outlined below:

Population: We will include studies on ST users (not e-cigarettes and other nicotine products-Electronic Nicotine Delivery Systems (ENDS)) of all age groups.

Interventions: Policy documents (FCTC and non-FCTC) within health and other sectors (commerce, finance, etc.) that describe the regulation or prohibition of ST products directly or indirectly will be considered eligible for RQ1. In addition, only those policies which have been implemented/enforced by the government will be included in the study for analysis. Policies which are at the planning stage (not implemented/enforced) until June 2020 will not be included. Specifically, we will include policies/legislations notified and enforced by any Ministry at the country-level. Documents related to the following policies will be included in the review:

FCTC Policies

- Pricing and taxation (Article 6)
- Product regulation (Article 9 and 10)
- Packaging and health warnings (Article 11)
- Education, communication, training, and public awareness (Article 12)
- Advertisement, promotion and sponsorship bans (Article 13)
- Cessation (Article 14)
- Illicit trade (Article 15)
- Sales to and by minors (Article 16)

Non-FCTC Policies

- Complete or partial ban on ST products (e.g. gutkha ban)
- Import ban
- Other policies mentioned to control ST (agriculture, environment etc.)

For peer review only - http://bmjopen.bmj.com/site/about/guidelines.xhtml

For RQ2, studies that report on the impact of the above-mentioned policies on ST related outcomes (see below) will be included.

We will exclude studies primarily looking at policies on harm reduction or switching between tobacco products. However, if the primary focus of studies is ST cessation then these studies will be considered eligible.

Comparators: Absence of intervention or usual care will be considered as comparators.

Outcomes: The outcomes to be included in the review will depend on the research questions. While RQ1 will be descriptive and will collate all policy measures undertaken in various countries, RQ2 will focus on studies reporting any of the following outcome measures in relation to specific policies (FCTC and non-FCTC).

a. Primary outcomes
   - Prevalence of ST use
   - ST quit rates
   - ST initiation
   - Health outcomes due to ST use
b. Intermediate outcomes
   - ST availability, affordability and accessibility
   - Change in knowledge, attitude, perception and norms related to ST use
   - Change in levels of chemical constituents
   - Noticing health warnings/advertisements
   - Recall of awareness campaigns
c. Reported unintended effects

Study Design: For RQ1, we will include policy documents, studies, editorials and commentaries. For RQ2, observational (cross-sectional/cohort/case-control) and intervention studies (randomized controlled trials/quasi experimental studies/economic evaluations) will be included in the review.

Databases

1. **For published studies**: The following global and regional databases will be searched to identify relevant published studies: Medline, EMBASE, PsycINFO, Cumulative Index to Nursing and Allied Health Literature (CINAHL), Scopus, EconLit, ISI Web of Science, Cochrane Library (CENTRAL), African Index Medicus (AIM), LILACS, Scientific

For peer review only - http://bmjopen.bmj.com/site/about/guidelines.xhtml

Electronic Library Online (SciELO), Global Medicus Index (which includes Index Medicus for the Eastern Mediterranean Region (IMEMR), Index Medicus for South-East Asia Region (IMSEAR), African Index Medicus (AIM) and Western Pacific Region Index Medicus (WPRIM)), and WHO Library Database (WHOLIS).

2. **For grey literature**: Grey literature search will be conducted on two different platforms as follows:
   a. Google search engine (first 100 hits) and target websites known within the research team such as WHO, Campaign for Tobacco Free Kids (CTFK), International clinical trials registry platform (WHO), EU Clinical Trials Register, ISRCTN, ProQuest, or those identified through Google search; and
   b. Country-specific government websites will be searched to collate policy documents (policies/laws/notifications/Acts) related to ST (FCTC and non-FCTC) in four South Asian countries (Bangladesh, India, Pakistan and Sri Lanka). The ministry websites searching will be restricted to South Asian countries only, given the timeframe and scope of the current study as well as due to language limitations. We will search websites of the Ministry of Health, Ministry of Commerce, Ministry of Finance, Ministry of Environment and any other ministries that might be relevant to either of the four countries.

Search Strategy

1. **For published studies**: The literature search will be conducted with key terms such as “smokeless tobacco”, “policies”, “legislation”, etc., with their synonyms (search string attached as Supplementary file 1. We will identify all ST control policies from around the world to address RQ1. For RQ2, from the same search, we will include studies that have estimated impact on ST use.

   References from other systematic reviews and chapters/textbooks will be assessed to identify potential studies, in addition to hand-searching reference lists and citations of included studies.

2. **For grey Literature:** Keywords including “smokeless tobacco” and “public policy” along with their relevant synonyms will be used to run the grey literature search. Key terms will be in alignment with the search terms of the literature review, however,

For peer review only - http://bmjopen.bmj.com/site/about/guidelines.xhtml

will be adapted to the specific contexts of the search engines (google search engine or ministry website search engine).

a. Google search engine: Google search engine will be used to search ST related documents published on the Internet. During our search, the first 100 hits or hits for results going back till the year 2005 (whichever comes first) will be screened for inclusion in the study. The results will be assessed using the title and the short description underneath, and all relevant records will be bookmarked in the browser and also entered into an Excel spreadsheet. Each entry will be made under the heading which has been titled as per the search strategy used. If the results include documents from India, Pakistan, Sri Lanka or Bangladesh, they will be saved in a separate excel spreadsheet to check for duplicates from the Ministry website searches. Newspaper articles will only be screened for identifying ST policies or their impact evaluation. Further, potential websites (such as WHO, CTFK) will be identified by Google search to identify ST policies or impact assessment studies related to ST policies. The dates of searching these websites will be documented in an excel spreadsheet. For the websites with a search engine, keywords will be used for searching the documents. For the websites without a search engine, hand searching will be conducted to identify relevant policies or impact assessment studies. The name, year and URL of these individual records within the websites would also be documented in the excel spreadsheet, as a sub-heading of the main website. All relevant entries that satisfy the inclusion criteria will then be screened.
b. Country-specific government websites: A search using keywords, "smokeless tobacco" and "public policy", with country-specific synonyms will be conducted on government websites across four SEAR countries (Bangladesh, India, Pakistan and Sri Lanka). All policy documents (policies, laws, legislations, acts and notifications) related to ST (FCTC and non-FCTC) will be searched on relevant ministry websites. Country level experts on the team who have experience in ST research will be consulted to select relevant policy documents from the searches. Only national level documents will be selected for this analysis as these policy guidelines prescribe the characteristics for sub-national policies. The title and summary of the policy documents will be screened. Details of potentially relevant records from all four countries will be added into an excel spreadsheet for full-text

For peer review only - http://bmjopen.bmj.com/site/about/guidelines.xhtml

screening. Relevant policy documents after full-text screening and de-duplication with google search results, will be included in the review.

To avoid duplication of the work and ensure comparable approaches, regular meetings to discuss search strategies among the team will be held.

Study records

Data management: Records from scientific databases will be imported into CADIMA (https://www.cadima.info/) and de-duplicated.

Study Selection: All the studies identified through database searching and policy documents identified through grey literature searches will be assessed by two reviewers independently for inclusion in the review based on predefined inclusion criteria (stage 1: titles and/or abstracts, stage 2: full text). Any disagreement will be resolved through mutual discussion and if needed, through a third reviewer.

Data extraction: Data extraction procedures will follow Preferred Reporting Items for Systematic Reviews and Meta-Analyses (PRISMA) guidelines. A standardised data extraction form will be used to extract data from the included policy documents and studies. The extracted information will include: study setting; location; year/s; population; participant demographics and baseline characteristics; details of the policies (policy specific to which Article of FCTC or whether beyond FCTC) and comparator conditions; study design; sample size and study completion rates; outcomes and times of measurement; information for assessment of the risk of bias and strength of evidence. Data extraction will be done by two reviewers independently. Missing data will be requested from study authors.

Quality assessment

Critical appraisal will be conducted only for academic publications literature, for which we will use the Effective Public Health Practice Project (EPHPP) "Quality Assessment Tool for Quantitative Studies".[20,21] The tool will assess study quality on eight domains: selection bias, study design, confounders, blinding, data collection methods, withdrawals and drop-outs, intervention integrity, analyses. It also gives an overall global rating of weak/moderate/strong evidence, which will be used for sensitivity analyses for RQ2.

For peer review only - http://bmjopen.bmj.com/site/about/guidelines.xhtml

Data Synthesis

For RQ1: Identified policies will be listed according to the focus of the intervention and the target population.

Focus of intervention: grouped as 'FCTC and non-FCTC' policies.

Target population: A further grouping by 'children/youth' and 'adults' will be applied.

Identified policies will be described using the TiDieR checklist for completion and understanding of the context; presented in a tabular form.[22] Conclusions will be drawn in relation to the review question and adequacy of description of the policies across the groupings (Tobacco specific and non-specific; children/youth and adults), summarised narratively to highlight gaps in existing policies for ST control.

For RQ2: We will conduct a meta-analysis on the primary outcomes (prevalence, quit rates, initiation, health outcome) if:

i) Ten or more studies are identified for the particular outcome and

ii) Heterogeneity in reported effects (I-squared) is less than 50% using STATA (version 16).

Key study characteristics (e.g., study design, population, location, risk of bias) will be presented in a tabular form. Summary effect measures will vary between the outcomes; for prevalence and initiation these will be the 'percentage change', and for the quit rates and health outcome these will be odds ratios. Effects for individual studies will be combined using random-effects models and presented as forest plots. Further, subgroups will be defined by:

i) study quality

ii) Focus of intervention (FCTC or non-FCTC), and

iii) Target population (children/youth and adults).

Poor overlap of confidence intervals (CIs) between individual studies and statistically significant Cochran's Q (a chi-squared test) will be considered as evidence of heterogeneity. To quantify the degree of inconsistency between the studies' results, the I - squared statistic (I-squared = 100% × (Q – df)/Q, where Q is Cochran's heterogeneity statistic and df, the degrees of freedom) will be computed for each outcome. We will assess the publication bias by using Begg's funnel plot by visual inspection for asymmetry.

For peer review only - http://bmjopen.bmj.com/site/about/guidelines.xhtml

Intermediate outcomes (where available for the identified policies) will be described and compared narratively across the tobacco specific and non-specific policies as well as by the target age of the population (children/youth and adults). The ST context and unintended consequences will also be synthesised narratively using a pre-designed conceptual framework on the major influences of the tobacco industry, governments and society on ST context (and their interactions); and the interaction between individual factors and ST use context to shape its uptake and consumption.

Context

We will extract any context related information from the papers on the following: physical, sociocultural, political (e.g. existence of FCTC and years of implementation) and economic aspects. We will also examine any unintended consequences of policies on switching from smoking to ST products, where data allows. We will use the framework (Figure 1), for a narrative synthesis of the contextual elements that are identified from our included studies.

Ethics and Dissemination Plan

As this study will be a review of published or publicly available data, there are no ethical concerns related to the involvement of humans or any other. Permission for ethics exemption of the review was obtained from the Centre for Chronic Disease Control's Institutional Ethics Committee, India (CCDC_IEC_06_2020; dated 16 April 2020). Data will be collected from publicly available scientific or grey literature through above-mentioned databases. On completion of the review, we will prepare a manuscript for publication in a peer-reviewed journal and present results in national and international conferences.

Patient and Public Involvement: No patients involved.

Contributors: AC and NJ wrote the first draft of the manuscript under MA's supervision. MK reviewed the statistical analysis plan. OD supervised the protocol development. All

For peer review only - http://bmjopen.bmj.com/site/about/guidelines.xhtml

authors contributed to the design of the study and gave critical inputs to drafting the protocol manuscript.

Funding statement: This work is funded by the UK's National Institute for Health Research (NIHR) [ASTRA (Grant Reference Number 17/63/76)]. The views expressed are those of the authors and not necessarily those of the NIHR or the Department of Health and Social Care.

Competing interests: None declared by any author

For peer review only

References

1. World Health Organization. WHO Framework Convention on Tobacco Control. Geneva; 2005.

For peer review only - http://bmjopen.bmj.com/site/about/guidelines.xhtml

2. Mehrotra R, Yadav A, Sinha DN, Parascandola M, John RM, Ayo-Yusuf O, et al. Smokeless tobacco control in 180 countries across the globe: call to action for full implementation of WHO FCTC measures. Vol. 20, The Lancet Oncology. Lancet Publishing Group; 2019. p. e208–17.

3. Sinha DN, Suliankatchi RA, Gupta PC, Thamarangsi T, Agarwal N, Parascandola M, et al. Global burden of all-cause and cause-specific mortality due to smokeless tobacco use: Systematic review and meta-analysis. Tob Control. 2018 Jan 1;27(1):35–42.

4. Siddiqi K, Lakshmi Vidyasagaran A, Readshaw A, Croucher R. A Policy Perspective on the Global Use of Smokeless Tobacco. 2017;

5. Siddiqi K, Shah S, Abbas SM, Vidyasagaran A, Jawad M, Dogar O, et al. Global burden of disease due to smokeless tobacco consumption in adults: analysis of data from 113 countries. BMC Med [Internet]. 2015 Dec 17 [cited 2019 Dec 12];13(1):194. Available from: http://bmcmedicine.biomedcentral.com/articles/10.1186/s12916-015-0424-2

6. IARC Working Group on the Evaluation of Carcinogenic Risk to Humans. IARC Monographs on the Evaluation of Carcinogenic Risks to Humans - Smokeless Tobacco and some Tobacco-speicific N-Nitrosamines. Vol. 89, Monograph. Lyon, France: International Agency for Research on Cancer; 2007.

7. Teo KK, Ounpuu S, Hawken S, Pandey M, Valentin V, Hunt D, et al. Tobacco use and risk of myocardial infarction in 52 countries in the INTERHEART study: a case-control study. Lancet. 2006 Aug 19;368(9536):647–58.

8. Niaz K, Maqbool F, Khan F, Bahadar H, Ismail Hassan F, Abdollahi M. Smokeless tobacco (paan and gutkha) consumption, prevalence, and contribution to oral cancer. Vol. 39, Epidemiology and health. 2017. p. e2017009.

9. Gupta PC, Subramoney S. Smokeless tobacco use and risk of stillbirth: A cohort study in Mumbai, India. Epidemiology [Internet]. 2006 Jan [cited 2020 Mar 19];17(1):47–51. Available from: http://www.ncbi.nlm.nih.gov/pubmed/16357594

10. Hossain MM, Rahman ME, Khan TH. Maternal smokeless tobacco use and adverse pregnancy outcome. Mymensingh Med J. 2014 Jan 1;23(1):46–51.

11. Suliankatchi RA, Sinha DN. The Human Cost of Tobacco Chewing Among Pregnant Women in India: A Systematic Review and Meta-analysis. J Obstet Gynecol India

For peer review only - http://bmjopen.bmj.com/site/about/guidelines.xhtml

[Internet]. 2016 Oct 7 [cited 2020 Mar 19];66(S1):161–6. Available from: http://link.springer.com/10.1007/s13224-015-0821-7

12. Levy DT, Mays D, Boyle RG, Tam J, Chaloupka FJ. The Effect of Tobacco Control Policies on US Smokeless Tobacco Use: A Structured Review. Vol. 20, Nicotine & tobacco research : official journal of the Society for Research on Nicotine and Tobacco. 2017. p. 3–11.
13. Tata Institute of Social Sciences, Ministry of Health and Family Welfare Government of India. Global Adult Tobacco Survey GATS 2 India. 2017;
14. Global Adult Tobacco Survey Bangladesh Factsheet [Internet]. 2017. Available from: http://bbs.portal.gov.bd/sites/default/files/files/bbs.portal.gov.bd/page/57def76a_aa3c_46e3_9f80_53732eb94a83/GATS_BAN_2017_Fact Sheet.pdf
15. Alpert HR, Vardavas CI, Chaloupka FJ, Vozikis A, Athanasakis K, Kyriopoulos I, et al. The recent and projected public health and economic benefits of cigarette taxation in Greece. Tob Control [Internet]. 2014 Sep [cited 2020 Mar 19];23(5):452–4. Available from: http://www.ncbi.nlm.nih.gov/pubmed/23467654
16. Siddiqi K, Mishu MP. Smokeless tobacco: Why does it need special attention? Respirology [Internet]. 2019 Aug 13 [cited 2019 Dec 12];24(8):720–1. Available from: https://onlinelibrary.wiley.com/doi/abs/10.1111/resp.13612
17. National Cancer Institute and Centers for Disease Control and Prevention. Smokeless Tobacco and Public Health: A Global Perspective. Bethesda, MD; 2014.
18. Australian Government Department of Health. Smoking and tobacco laws in Australia [Internet]. [cited 2020 Jun 5]. Available from: https://www.health.gov.au/health-topics/smoking-and-tobacco/about-smoking-and-tobacco/smoking-and-tobacco-laws-in-australia
19. Pimple S, Gunjal S, Mishra GA, Pednekar MS, Majmudar P, Shastri SS. Compliance to Gutka ban and other provisons of COTPA in Mumbai. Indian J Cancer. 2014 Oct 1;51(5):S60–6.
20. Thomas BH, Ciliska D, Dobbins M, Micucci S. A process for systematically reviewing the literature: Providing the research evidence for public health nursing interventions. Worldviews Evidence-Based Nurs [Internet]. 2004 Sep [cited 2020 Mar 19];1(3):176–84. Available from:

For peer review only - http://bmjopen.bmj.com/site/about/guidelines.xhtml

http://www.ncbi.nlm.nih.gov/pubmed/17163895

21. Armijo-Olivo S, Stiles CR, Hagen NA, Biondo PD, Cummings GG. Assessment of study quality for systematic reviews: A comparison of the Cochrane Collaboration Risk of Bias Tool and the Effective Public Health Practice Project Quality Assessment Tool: Methodological research. J Eval Clin Pract [Internet]. 2012 Feb [cited 2020 Mar 19];18(1):12–8. Available from: http://www.ncbi.nlm.nih.gov/pubmed/20698919

22. Hoffmann TC, Glasziou PP, Boutron I, Milne R, Perera R, Moher D, et al. Better Reporting of Interventions: Template for Intervention Description and Replication (TIDieR) Checklist and Guide. BMJ [Internet]. 2014 [cited 2020 Jul 9];348. Available from: http://www.bmj.com/content/348/bmj.g1687

For peer review only

For peer review only - http://bmjopen.bmj.com/site/about/guidelines.xhtml

Figure 1. The four main components of ST context; the major influences of the tobacco industry, governments and society on ST context (and their interactions); and the interaction between individual factors and ST use context to shape its uptake and consumption.[26]

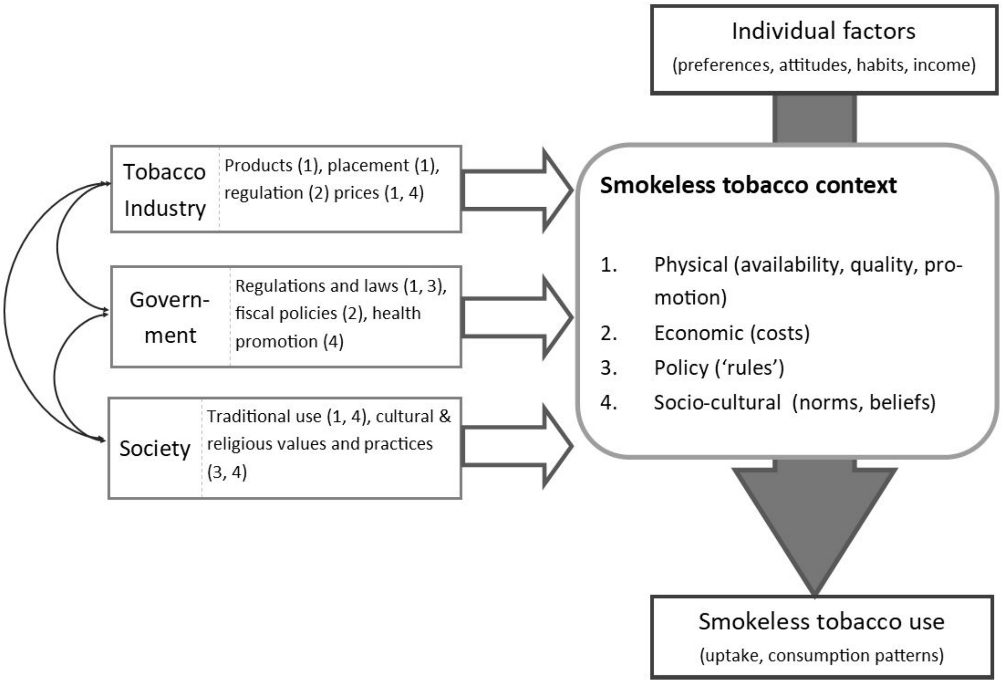

Database: Ovid MEDLINE(R) ALL <1946 to April 14, 2020>
Search Strategy:
----
1 Tobacco, Smokeless/ (3622)
2 (tobacco adj4 (SLT or ST)).ti,ab,kw. (543)
3 (tobacco adj4 (chew$ or dipping or eating or loose-leaf or oral or plug or sneeze or tablet$ or toothpaste or twist$)).ti,ab,kw. (2647)
4 (al-shammah or chemma or chimo or el-shama or ghutkha or gudakhu or gul or gutkha or gutka or iqmik or jarda or khaini or khiwam or kiwan or makla or mawa or mishri or nass or nasvay or naswar or neffa or nufha or paan or quid or sadapata or shammah or snuff or snuif or snus or taaba or tambakoo or tenfeha or tombak or tombol or toombak or tuibur or vizapatta or zarda).ti,bt,ab,kw. (4355)
5 (pan adj4 (tobacco or smokeless)).ti,ab,kw. (75)
6 (nas adj4 (tobacco or smokeless)).ti,ab,kw. (5)
7 1 or 2 or 3 or 4 or 5 or 6 (8157)
8 Public Policy/ or Government Regulation/ or Social Control, Formal/ or Taxation/ (62112)
9 (policies or policy or regulation$ or rule$ or law$ or legislation or tax or taxation or taxes).ti,ab. (1379493)
10 (strateg$ or program$).ti,ab. (1835226)
11 (ban or bans or banning or banned).ti,ab. (14063)
12 Advertising/ or Commerce/ or Marketing/ or Product Packaging/ (44071)
13 (price or prices or pricing or packaging or advertis$ or marketing or sponsor$).ti,ab. (118491)
14 Health Education/ or Health Promotion/ or Mass Media/ (134812)
15 (health warning$ or campaign$ or labelling or label or labels).ti,ab. (213540)
16 (product adj2 regulat$).ti,ab. (1080)
17 (content adj2 disclos$).ti,ab. (65)
18 illicit trade.ti,ab. (118)
19 ((sale$ or selling or sold) adj3 (minor$ or child$)).ti,ab. (390)
20 Smoking Cessation/ (28128)
21 8 or 9 or 10 or 11 or 12 or 13 or 14 or 15 or 16 or 17 or 18 or 19 or 20 (3456116)
22 7 and 21 (2199)
23 (smokeless tobacco adj3 (cessation or prevent$ or stop$)).ti,ab. (127)
24 22 or 23 (2256)
25 neonatal abstinence syndrome.ti,ab. (916)
26 NASS Patient Satisfaction Index.ti,ab. (5)
27 Normal Astacus saline.ti,ab. (1)
28 naphthenic acid.ti,ab. (136)
29 nicotianamine synthase.ti,ab. (82)
30 25 or 26 or 27 or 28 or 29 (1140)
31 24 not 30 (2253)
32 limit 31 to yr="2005 -Current" (1601)

****
<1>
UI - 32279158

For peer review only

TI - Baseline characteristics of American Indian smokeless tobacco users participating in two pilot cessation studies.
SO - Journal of Community Health. 2020 Apr 11
AU - Lewis CS
AU - Nazir N
AU - Daley SM
AU - Pacheco J
AU - Goeckner RT
AU - Hale JW
AU - Gunville JA
AU - Rahman F
AU - Choi WS
AU - Daley CM
FA - Lewis, Charley S
FA - Nazir, Niaman
FA - Daley, Sean M
FA - Pacheco, Joseph
FA - Goeckner, Ryan T
FA - Hale, Jason W
FA - Gunville, Jordyn A
FA - Rahman, Fatima
FA - Choi, Won S
FA - Daley, Christine Makosky
PT - Journal Article

For peer review only

PRISMA-P (Preferred Reporting Items for Systematic review and Meta-Analysis Protocols) 2015 checklist: recommended items to address in a systematic review protocol*

Section and topic	Item No	Checklist item	Status
**ADMINISTRATIVE INFORMATION**			
Title:			
Identification	1a	Identify the report as a protocol of a systematic review	✓
Update	1b	If the protocol is for an update of a previous systematic review, identify as such	Not Applicable
Registration	2	Mention Registration Number (such as for PROSPERO)	CRD42020191946
Authors:			
Contact	3a	Provide name, institutional affiliation, e-mail address of all protocol authors; provide physical mailing address of corresponding author	✓
Contributions	3b	Describe contributions of protocol authors and identify the guarantor of the review	✓
Amendments	4	If the protocol represents an amendment of a previously completed or published protocol, identify as such and list changes; otherwise, state plan for documenting important protocol amendments	Not Applicable
Support:			
Sources	5a	Indicate sources of financial or other support for the review	✓
Sponsor	5b	Provide name for the review funder and/or sponsor	✓
Role of sponsor or funder	5c	Describe roles of funder(s), sponsor(s), and/or institution(s), if any, in developing the protocol	✓
**INTRODUCTION**			
Rationale	6	Describe the rationale for the review in the context of what is already known	✓
Objectives	7	Provide an explicit statement of the question(s) the review will address with reference to participants, interventions, comparators, and outcomes (PICO)	✓
**METHODS**			
Eligibility criteria	8	Specify the study characteristics (such as PICO, study design, setting, time frame) and report characteristics (such as years considered, language, publication status) to be used as criteria for eligibility for the review	✓
Information sources	9	Describe all intended information sources (such as electronic databases, contact with study authors, trial registers or other grey literature sources) with planned dates of coverage	✓

For peer review only

Search strategy	10	Present draft of search strategy to be used for at least one electronic database, including planned limits, such that it could be repeated	✓
Study records:			
Data management	11a	Describe the mechanism(s) that will be used to manage records and data throughout the review	✓
Selection process	11b	State the process that will be used for selecting studies (such as two independent reviewers) through each phase of the review (that is, screening, eligibility and inclusion in meta-analysis)	✓
Data collection process	11c	Describe planned method of extracting data from reports (such as piloting forms, done independently, in duplicate), any processes for obtaining and confirming data from investigators	✓
Data items	12	List and define all variables for which data will be sought (such as PICO items, funding sources), any pre-planned data assumptions and simplifications	✓
Outcomes and prioritization	13	List and define all outcomes for which data will be sought, including prioritization of main and additional outcomes, with rationale	✓
Risk of bias in individual studies	14	Describe anticipated methods for assessing risk of bias of individual studies, including whether this will be done at the outcome or study level, or both; state how this information will be used in data synthesis	✓
Data synthesis	15a	Describe criteria under which study data will be quantitatively synthesised	✓
	15b	If data are appropriate for quantitative synthesis, describe planned summary measures, methods of handling data and methods of combining data from studies, including any planned exploration of consistency (such as I^2, Kendall's τ)	✓
	15c	Describe any proposed additional analyses (such as sensitivity or subgroup analyses, meta-regression)	
	15d	If quantitative synthesis is not appropriate, describe the type of summary planned	✓ (described for narrative synthesis)
Meta-bias(es)	16	Specify any planned assessment of meta-bias(es) (such as publication bias across studies, selective reporting within studies)	✓
Confidence in cumulative evidence	17	Describe how the strength of the body of evidence will be assessed (such as GRADE)	✓

*** It is strongly recommended that this checklist be read in conjunction with the PRISMA-P Explanation and Elaboration (cite when available) for important clarification on the items. Amendments to a review protocol should be tracked and dated. The copyright for PRISMA-P (including checklist) is held by the PRISMA-P Group and is distributed under a Creative Commons Attribution Licence 4.0.**

For peer review only

From: Shamseer L, Moher D, Clarke M, Ghersi D, Liberati A, Petticrew M, Shekelle P, Stewart L, PRISMA-P Group. Preferred reporting items for systematic review and meta-analysis protocols (PRISMA-P) 2015: elaboration and explanation. BMJ. 2015 Jan 2;349(jan02 1):g7647.

For peer review only

BMJ Open

The global impact of tobacco control policies on smokeless tobacco use: a systematic review protocol

Journal:	*BMJ Open*
Manuscript ID	bmjopen-2020-042860.R1
Article Type:	Protocol
Date Submitted by the Author:	15-Oct-2020

Complete List of Authors:	Arora, Monika; HRIDAY, ; Public Health Foundation of India, Health Promotion<br>Chugh, Aastha; HRIDAY<br>Jain, Neha; Public Health Foundation of India,<br>Mishu, Masuma; University of York Department of Health Sciences, Health Sciences<br>Boeckmann, Melanie; Bielefeld University, School of Public Health<br>Dahanayake, Suranji; University of York Department of Health Sciences, Health Sciences<br>Eckhardt, Jappe; University of York, Politics<br>Forberger, Sarah; Leibniz Institute for Prevention Research ,<br>Huque, Rumana; University of Dhaka, Economics<br>Kanaan, Mona; University of York, Health Sciences<br>khan, Zohaib; Khyber Medical University, ;<br>Mehrotra, Ravi; National Institute of Cancer Prevention and Research, Department of Health Research<br>Rahman, Muhammad; Federation University Australia, School of Health; La Trobe University,<br>Readshaw, Anne; University of York Department of Health Sciences, Health Sciences; University ofYork<br>Sheikh, Aziz; University of Edinburgh, Division of Community Health Sciences<br>Siddiqi, Kamran; University of York, UK, Institute of Health Sciences<br>Vidyasagaran, Aishwarya; University of York Department of Health Sciences, Health Sciences<br>Dogar, Omara; University of York, Health Sciences

**Primary Subject Heading**:	Health policy
Secondary Subject Heading:	Global health, Public health, Smoking and tobacco
Keywords:	Health policy < HEALTH SERVICES ADMINISTRATION & MANAGEMENT, Protocols & guidelines < HEALTH SERVICES ADMINISTRATION & MANAGEMENT, International health services < HEALTH SERVICES ADMINISTRATION & MANAGEMENT, Public health < INFECTIOUS DISEASES

SCHOLARONE™
Manuscripts

I, the Submitting Author has the right to grant and does grant on behalf of all authors of the Work (as defined in the below author licence), an exclusive licence and/or a non-exclusive licence for contributions from authors who are: i) UK Crown employees; ii) where BMJ has agreed a CC-BY licence shall apply, and/or iii) in accordance with the terms applicable for US Federal Government officers or employees acting as part of their official duties; on a worldwide, perpetual, irrevocable, royalty-free basis to BMJ Publishing Group Ltd ("BMJ") its licensees and where the relevant Journal is co-owned by BMJ to the co-owners of the Journal, to publish the Work in this journal and any other BMJ products and to exploit all rights, as set out in our licence.

The Submitting Author accepts and understands that any supply made under these terms is made by BMJ to the Submitting Author unless you are acting as an employee on behalf of your employer or a postgraduate student of an affiliated institution which is paying any applicable article publishing charge ("APC") for Open Access articles. Where the Submitting Author wishes to make the Work available on an Open Access basis (and intends to pay the relevant APC), the terms of reuse of such Open Access shall be governed by a Creative Commons licence – details of these licences and which Creative Commons licence will apply to this Work are set out in our licence referred to above.

Other than as permitted in any relevant BMJ Author's Self Archiving Policies, I confirm this Work has not been accepted for publication elsewhere, is not being considered for publication elsewhere and does not duplicate material already published. I confirm all authors consent to publication of this Work and authorise the granting of this licence.

For peer review only

Title: The global impact of tobacco control policies on smokeless tobacco use: a systematic review protocol

Authors: Monika Arora[1, 2], Aastha Chugh[1], Neha Jain[2], Masuma Pervin Mishu[3], Melanie Boeckmann[3,4], Suranji Dahanayake[3,5], Jappe Eckhardt[3], Sarah Forberger[6], Rumana Huque[7], Mona Kanaan[3], Zohaib Khan[8], Ravi Mehrotra[9], Muhammad Aziz Rahman[10,11], Anne Readshaw[3], Aziz Sheikh[12], Kamran Siddiqi[3], Aishwarya Vidyasagaran[3], Omara Dogar[3,12]

Affiliations: [1]HRIDAY (India), [2]Public Health Foundation of India (India), [3]University of York (United Kingdom),[4] Bielefeld University School of Public Health (Germany), [5] Ministry of Health, Nutrition and Indigenous Medicine (Sri Lanka), [6]Leibniz Institute For Prevention Research and Epidemiology, BIPS (Germany), [7]ARK Foundation (Bangladesh), [8]Khyber Medical University (Pakistan), [9]Indian Council of Medical Research (India),[10]Federation University Australia (Australia), [11]La Trobe University (Australia), [12]University of Edinburgh (United Kingdom)

Acknowledgements: Ian Kellar (University of Leeds, United Kingdom), Subhash Pokhrel (Brunel University, United Kingdom), ASTRA Global Health Research Group (https://www.york.ac.uk/healthsciences/research/public-health/projects/astra/)

Corresponding Author: J.-Prof. Dr. Melanie Boeckmann, Bielefeld University School of Public Health, Department of Environment and Health, 33615 Bielefeld, Germany, Email: Melanie.boeckmann@uni-bielefeld.de

Keywords: Tobacco control, Smokeless tobacco, policies, impact, systematic review

Word Count: 3497 (excluding figure and references)

For peer review only - http://bmjopen.bmj.com/site/about/guidelines.xhtml

Abstract

Introduction: Smokeless tobacco (ST) was consumed by 356 million people globally in 2017. Recent evidence shows that ST consumption is responsible for an estimated 652,494 all-cause deaths across the globe annually. The WHO FCTC was negotiated in 2003 and ratified in 2005 to implement effective tobacco control measures. While the policy measures enacted through various tobacco control laws have been effective in reducing the incidence and prevalence of smoking, the impact of ST-related policies (within WHO FCTC and beyond) on ST use is under-researched and not collated.

Methods and Analysis: A systematic review will be conducted to collate all available ST related policies implemented across various countries and assess their impact on ST use. The following databases will be searched: Medline, EMBASE , PsycINFO, Cumulative Index to Nursing and Allied Health Literature (CINAHL), Scopus, EconLit, ISI Web of Science, Cochrane Library (CENTRAL), African Index Medicus (AIM), LILACS, Scientific Electronic Library Online (SciELO), Index Medicus for the Eastern Mediterranean Region (IMEMR), Index Medicus for South-East Asia Region (IMSEAR), Western Pacific Region Index Medicus (WPRIM), and WHO Library Database (WHOLIS); as well as Google search engine and country-specific government websites. All ST related policy documents (FCTC and non-FCTC) will be included. Results will be limited to literature published since 2005 in English and regional languages (Bengali, Hindi and Urdu). Two reviewers will independently employ two-stage screening to determine inclusion. The Effective Public Health Practice Project (EPHPP)'s "Quality Assessment Tool for Quantitative Studies" will be used to record ratings of quality and risk of bias among studies selected for inclusion. Data will be extracted using a standardised form. Meta-analysis and narrative synthesis will be used.

Ethics and Dissemination: Permission for ethics exemption of the review was obtained from the Centre for Chronic Disease Control's Institutional Ethics Committee, India (CCDC_IEC_06_2020; dated 16 April 2020). The results will be disseminated through publications in a peer-reviewed journal and will be presented in national and international conferences.

PROSPERO Registration Number: CRD42020191946

For peer review only

Article Summary

Strengths and limitations of the study:

- This is a protocol for a systematic review that is a comprehensive global review of all available ST related policies.
- The review will include all available ST related policies (FCTC and non-FCTC policies). It will also include ST policies notified/enforced by various government bodies such as Ministry of Health, Ministry of Finance, Ministry of Commerce etc..
- This review is the first attempt to search for ST related policies globally and assess their impact systematically across academic databases and country-specific government websites.
- Studies and policy documents in English and regional languages for which expertise is available in the team (Bangla, Hindi, and Urdu) will be included in the review. Due to limited resources, documents in other languages will not be included.

For peer review only

For peer review only - http://bmjopen.bmj.com/site/about/guidelines.xhtml

Introduction

Tobacco products are broadly classified into two categories – smoked and smokeless tobacco (ST). The World Health Organization's Framework Convention on Tobacco Control (WHO FCTC) defines ST as "tobacco that is consumed in un-burnt form either orally or nasally".[1] A wide range of ST products are manufactured and consumed worldwide, some of which are snus, pan masala, gutkha, mawa, khaini, zarda, mishri, dry snuff, moist snuff etc. ST was consumed by 356 million people globally in 2017.[2] Cumulatively, it is estimated that, globally, a total of 652,494 people die from ST attributed diseases annually.[3] It is used in about 116 countries around the world[4], and a vast majority of users reside in South and South-East Asia, which bears more than 85% of the burden of diseases due to ST use.[5] The practice of ST use is culturally influenced and widespread in this region, where one-third of tobacco is consumed in the smokeless form.[6] This widespread use of ST products is a cause for alarm, considering that they have been known to contain more than 30 carcinogens.[6] The INTERHEART study, a large case-control study published in 2006 and undertaken in 52 countries, found that 4.7 million DALYs were lost and 204,309 people died from coronary heart diseases caused by ST consumption.[7] ST use leads to oral sub-mucous fibrosis and cancers of the oral cavity.[8] Pregnant women using ST have shown a threefold increased risk of stillbirth and two- to threefold increased risk of having low-birth-weight babies.[9–11]

The WHO FCTC was negotiated in 2003, and ratified in 2005, with the aim of providing guidelines to implement effective tobacco control measures.[1] Currently, there are 183 country-level signatories to this treaty. Though the menace of ST use is considered to be relevant to fewer countries and regions than smoking, it is evident that there has been an increase in the prevalence of ST use, even in economically-developed countries.[12] However, the situation is worse in low- and middle-income countries (LMICs), such as India, where the prevalence of ST use is higher than smoking.[13] While 10.7% of all the adults in India are smokers, 21.4% are current ST users.[13] Similarly in Bangladesh, while 18% of all adults are smokers, 20.6% are ST users.[14]

Furthermore, while the policies described under FCTC have been successful in decreasing the incidence and prevalence of smoking, there is insufficient evidence on the impact of the same on ST use.[2] For example, taxation and smoke-free policies (as described in Article 6 of the FCTC) have been successful in reducing the prevalence of smoking in countries such as Greece.[15] However, the impact of these policies on ST use is not widely known, nor have they been systematically collated. This could be attributed to the fact that ST has not been given the same importance as smoking while implementing these policies. In addition, these policies have often been based on evidence gathered

For peer review only - http://bmjopen.bmj.com/site/about/guidelines.xhtml

from research on cigarettes conducted in high-income countries, and hence the translation of the same policies for the control of ST in LMICs has not been contextualised.[16] For instance, mass media campaigns for raising awareness on ST have been highlighted by very few countries, and sale of ST products to minors is illegal only in a handful of the countries.[2,17] In addition, second hand impact of ST use (such as diseases linked to spitting) and its adverse environmental impacts due to disposal of small sachets of ST products) do not invoke any public health response. The burden of ST is now being realized in some developed countries too. For example, Australia recently imposed a ban on import, production, sale, distribution and advertisement of ST products, including chewing tobacco, oral snuff, tobacco paste and powder.[18] On the other hand, in the United States of America, the Food and Drug Administration (FDA) now considers ST as a modified risk tobacco product, under their Family Smoking Prevention and Tobacco Control Act 2009.[12] This change in policy scenario has been neither persistent nor uniform across the world. Countries like India and Sri Lanka have gone beyond FCTC to include the prohibition of some (e.g. gutka) or all ST products.[19] The impact of FCTC policies, as well as country-specific tobacco control laws, on the consumption of ST is under-researched and not collated.

Aims and Objectives

The study aims to undertake a systematic review to collate all policies implemented across countries globally relevant to the control of ST products and assess their impact on ST use.

Specific review questions include:

Review Question 1 (RQ1): What are the existing policies and legislation (FCTC and non-FCTC) related to the control of ST products across various countries globally?

Review Question 2 (RQ2): What is the impact of these policies on controlling ST use, including its uptake, quit and reduction?

Methodology

The study is a systematic review which will consider all relevant studies from 2005 onwards (after the WHO FCTC came into force) without any geographical restriction. Articles in English and for which linguistic expertise is available within the team (Bengali, Hindi and Urdu) will be included. Our study will include published articles (including commentaries and editorials) searched through scientific databases. Policy documents (policies/laws/notifications/Acts related to ST) identified through grey literature searches will also be included in the review. Systematic reviews will be excluded because we expect to cover most of the relevant literature through our searches and a review

For peer review only - http://bmjopen.bmj.com/site/about/guidelines.xhtml

of systematic reviews will be beyond the scope of the study. Studies reporting at least one of the outcomes (detailed below) in relation to at least one policy of interest will be eligible for inclusion. We will identify studies using keywords such as ''smokeless tobacco'', ''public policies'', etc., with synonyms, and based on the PICOS criteria outlined below:

Population: We will include studies on ST users (not e-cigarettes and other nicotine products-Electronic Nicotine Delivery Systems (ENDS)) of all age groups.

Interventions: Policy documents (FCTC and non-FCTC) within health and other sectors (commerce, finance, etc.) that describe the regulation or prohibition of ST products directly or indirectly will be considered eligible for RQ1. In addition, only those policies which have been implemented/enforced by the government will be included in the study for analysis. Policies which are at the planning stage (not implemented/enforced) until June 2020 will not be included. Specifically, we will include policies/legislations notified and enforced by governmental bodies such as Ministry of Health, Ministry of Finance etc., at the country-level. Documents related to the following policies will be included in the review:

FCTC Policies

- Pricing and taxation (Article 6)
- Product regulation (Article 9 and 10)
- Packaging and health warnings (Article 11)
- Education, communication, training, and public awareness (Article 12)
- Advertisement, promotion and sponsorship bans (Article 13)
- Cessation (Article 14)
- Illicit trade (Article 15)
- Sales to and by minors (Article 16)

Non-FCTC Policies

- Complete or partial ban on ST products (e.g. gutkha ban)
- Import ban
- Other policies mentioned to control ST (agriculture, environment etc.)

For peer review only - http://bmjopen.bmj.com/site/about/guidelines.xhtml

For RQ2, studies that report on the impact of the above-mentioned policies on ST related outcomes (see below) will be included.

We will exclude studies primarily looking at policies on harm reduction or switching between tobacco products. However, if the primary focus of studies is ST cessation then these studies will be considered eligible.

Comparators: Absence of intervention or usual care will be considered as comparators.

Outcomes: The outcomes to be included in the review will depend on the research questions. While RQ1 will be descriptive and will collate all policy measures undertaken in various countries, RQ2 will focus on studies reporting any of the following outcome measures in relation to specific policies (FCTC and non-FCTC).

a. Primary outcomes
   - Percentage change in prevalence of ST use
   - Rates of quitting ST (proportion of those who stopped out of total intervened)
   - Percentage change in ST initiation (from never used to ever used)
   - Change in health outcomes for cancers and cardiovascular diseases attributable to ST (ST-related disease burden is substantial for these conditions, as noted in a recent study).[20]

b. Intermediate outcomes
   - ST availability, affordability and accessibility
   - Change in knowledge, attitude, perception and norms related to ST use
   - Change in levels of chemical constituents
   - Noticing health warnings/advertisements
   - Recall of awareness campaigns

c. Reported unintended effects

Study Design: For RQ1, we will include policy documents, studies, editorials and commentaries. For RQ2, observational (cross-sectional/cohort/case-control) and intervention studies (randomized controlled trials/quasi experimental studies/economic evaluations) will be included in the review.

Databases

1. **For published studies**: The following global and regional databases will be searched to identify relevant published studies: Medline, EMBASE, PsycINFO, Cumulative Index to Nursing and

For peer review only - http://bmjopen.bmj.com/site/about/guidelines.xhtml

Allied Health Literature (CINAHL), Scopus, EconLit, ISI Web of Science, Cochrane Library (CENTRAL), African Index Medicus (AIM), LILACS, Scientific Electronic Library Online (SciELO), Global Medicus Index (which includes Index Medicus for the Eastern Mediterranean Region (IMEMR), Index Medicus for South-East Asia Region (IMSEAR), African Index Medicus (AIM) and Western Pacific Region Index Medicus (WPRIM)), and WHO Library Database (WHOLIS).

2. **For grey literature**: Grey literature search will be conducted on two different platforms as follows:
   a. Google search engine (first 100 hits) and target websites known within the research team such as WHO, Campaign for Tobacco Free Kids (CTFK), International clinical trials registry platform (WHO), EU Clinical Trials Register, ISRCTN, ProQuest, or those identified through Google search; and
   b. Country-specific government websites will be searched to collate policy documents (policies/laws/notifications/Acts) related to ST (FCTC and non-FCTC) in four South Asian countries (Bangladesh, India, Pakistan and Sri Lanka). The ministry websites searching will be restricted to South Asian countries only, given the timeframe and scope of the current study as well as due to language limitations. We will search websites of the Ministry of Health, Ministry of Commerce, Ministry of Finance, Ministry of Environment and governmental bodies that might be relevant to either of the four countries.

Search Strategy

1. **For published studies**: The literature search will be conducted with key terms such as "smokeless tobacco", "policies", "legislation", etc., with their synonyms (search string attached as Supplementary file 1). We will identify all ST control policies from around the world to address RQ1. For RQ2, from the same search, we will include studies that have estimated impact on ST use.

   References from other systematic reviews and chapters/textbooks will be assessed to identify potential studies, in addition to hand-searching reference lists and citations of included studies.

2. **For grey Literature:** Keywords including "smokeless tobacco" and "public policy" along with their relevant synonyms will be used to run the grey literature search. Key terms will be in alignment with the search terms of the literature review, however, will be adapted to the specific contexts of the search engines (google search engine or ministry website search engine).

For peer review only - http://bmjopen.bmj.com/site/about/guidelines.xhtml

a. Google search engine: Google search engine will be used to search ST related documents published on the Internet. During our search, the first 100 hits or hits for results going back till the year 2005 (whichever comes first) will be screened for inclusion in the study. The results will be assessed using the title and the short description underneath, and all relevant records will be bookmarked in the browser and also entered into an Excel spreadsheet. Each entry will be made under the heading which has been titled as per the search strategy used. If the results include documents from India, Pakistan, Sri Lanka or Bangladesh, they will be saved in a separate excel spreadsheet to check for duplicates from the Ministry website searches. Newspaper articles will only be screened for identifying ST policies or their impact evaluation. Further, potential websites (such as WHO, CTFK) will be identified by Google search to identify ST policies or impact assessment studies related to ST policies. The dates of searching these websites will be documented in an excel spreadsheet. For the websites with a search engine, keywords will be used for searching the documents. For the websites without a search engine, hand searching will be conducted to identify relevant policies or impact assessment studies. The name, year and URL of these individual records within the websites would also be documented in the excel spreadsheet, as a sub-heading of the main website. All relevant entries that satisfy the inclusion criteria will then be screened.

b. Country-specific government websites: A search using keywords, ‘‘smokeless tobacco” and “public policy”, with country-specific synonyms will be conducted on government websites across four SEAR countries (Bangladesh, India, Pakistan and Sri Lanka). All policy documents (policies, laws, legislations, acts and notifications) related to ST (FCTC and non-FCTC) will be searched on relevant ministry websites. Country level experts on the team who have experience in ST research will be consulted to select relevant policy documents from the searches. Only national level documents will be selected for this analysis as these policy guidelines prescribe the characteristics for sub-national policies. The title and summary of the policy documents will be screened. Details of potentially relevant records from all four countries will be added into an excel spreadsheet for full-text screening. Relevant policy documents after full-text screening and de-duplication with google search results, will be included in the review.

To avoid duplication of the work and ensure comparable approaches, regular meetings to discuss search strategies among the team will be held.

For peer review only

Study records

Data management: Records from scientific databases will be imported into CADIMA (https://www.cadima.info/) and de-duplicated.

Study Selection: All the studies identified through database searching and policy documents identified through grey literature searches will be assessed by two reviewers independently for inclusion in the review based on predefined inclusion criteria (stage 1: titles and/or abstracts, stage 2: full text). Any disagreement will be resolved through mutual discussion and if needed, through a third reviewer.

Data extraction: Data extraction procedures will follow Preferred Reporting Items for Systematic Reviews and Meta-Analyses (PRISMA) guidelines (see also the PRISMA-P checklist in Supplementary file 2). A standardised data extraction form (attached as Supplementary File 3) will be used to extract data from the included policy documents and studies. The extracted information will include: study setting; location; year/s; population; participant demographics and baseline characteristics; details of the policies (policy specific to which Article of FCTC or whether beyond FCTC) and comparator conditions; study design; sample size and study completion rates; outcomes and times of measurement; information for assessment of the risk of bias and strength of evidence. Data extraction will be done by two reviewers independently. Missing data will be requested from study authors.

Quality assessment

Critical appraisal will be conducted only for academic publications literature, for which we will use the Effective Public Health Practice Project (EPHPP) "Quality Assessment Tool for Quantitative Studies".[21,22] The tool will assess study quality on eight domains: selection bias, study design, confounders, blinding, data collection methods, withdrawals and drop-outs, intervention integrity, analyses. It also gives an overall global rating of weak/moderate/strong evidence, which will be used for sensitivity analyses for RQ2.

Data Synthesis

For RQ1: Identified policies will be listed according to the focus of the intervention and the target population.

Focus of intervention: grouped as 'FCTC and non-FCTC' policies.

For peer review only - http://bmjopen.bmj.com/site/about/guidelines.xhtml

Target population: A further grouping by 'children/youth' and 'adults' will be applied.

Identified policies will be described using the TiDieR checklist for completion and understanding of the context; presented in a tabular form.[23] Conclusions will be drawn in relation to the review question and adequacy of description of the policies across the groupings (Tobacco specific and non-specific; children/youth and adults), summarised narratively to highlight gaps in existing policies for ST control.

For RQ 2: Key study characteristics (e.g., study design, population, location, risk of bias) will be presented in a tabular form. We will conduct a meta-analysis on the primary outcomes, if:
i) 2 or more studies are identified for the particular outcome, and
ii) Heterogeneity in reported effects (I-squared) is less than 50%[24]

Meta-analyses will be conducted using a fixed-effect model, unless there is evidence of between-study heterogeneity.[25] Where heterogeneity is >=50% we will explore possible explanations using sub-group analysis, and will not report a pooled estimate of the effect. Heterogeneity (if more than 50% for any specific outcome) will be explored visually using tables and forest plots, by comparing the effect sizes of studies grouped according to potential effect modifiers. These include:

1. Focus of intervention (e.g. FCTC or non-FCTC, etc.);
2. Target population (e.g. children, youth, adults, elderly, etc.)
3. Quality of studies.

We will assess the publication bias by using Begg's funnel plot by visual inspection for asymmetry. Intermediate outcomes (where available for the identified policies) will be described and compared narratively across the tobacco specific and non-specific policies as well as by the target age of the population (children/youth and adults). The ST context and unintended consequences will also be synthesised narratively using a pre-designed conceptual framework on the major influences of the tobacco industry, governments and society on ST context (and their interactions); and the interaction between individual factors and ST use context to shape its uptake and consumption.

Context

We will extract any context related information from the papers on the following: physical, sociocultural, political (e.g. existence of FCTC and years of implementation) and economic aspects. We will also examine any unintended consequences of policies on switching from smoking to ST

For peer review only - http://bmjopen.bmj.com/site/about/guidelines.xhtml

products, where data allows. We will use the framework (Figure 1), for a narrative synthesis of the contextual elements that are identified from our included studies.

The INFORMAS framework[26] was used for contextualising the basis of this study because it is one of the most frequently used frameworks covering the complex interaction between industry, government and society. It is highly evidence-based but equally flexible and can be adapted to other lifestyles such as ST consumption. In addition, it helps to identify the key factors that play a role in explaining the behavior of individuals and the population and to find ways to modify population behavior. Although it is not a system map, it can be used as a basis for identifying the main influences. It also allows comparisons to be made between countries and between other areas of research concerned with healthy lifestyles, such as obesity prevention and physical activity. It also allows the transfer of results and helps to identify context-specific and best practice approaches and supports the development and design of policies based on national context.

The ST context framework follows the socio-behavioural theory and a basic premise of this theory is that people learn not only through their own experiences, but also by observing the actions of others and the results of those actions.[27] This is particularly relevant to ST use, which unlike individual smoking behaviour in combustible tobacco forms, is a socio-traditionally influenced behaviour.[28,29] ST consumption is unique in certain regions, such as South Asia, not only because of its traditional heritage and socio-cultural myths that promote consumption but also the variety of products from homemade or small scale production to imported licenced as well as illegal products that are logistically and politically challenging to regulate. Individuals, with their personal factors such as habits, preferences, education and income, interact with the context that determine their use of these products.

The context of ST and each of its component dimensions - physical (e.g. availability, quality, promotion), economic (e.g. costs, affordability), policy (e.g. laws, regulations and accessibility) and socio-cultural (e.g. norms and beliefs) - has substantial impact on uptake and consumption. Tobacco industry, governments and society can influence and shape these dimensions of ST context, thus playing a vital role in the uptake and consumption patterns of individuals. These external factors not only interact with each other but also interact with the individuals preferences, attitudes, habits and income etc. to shape their ST use behaviour. For example, governments, at international, national and local levels, through their policies, laws and regulations, provide the ‘rules’ within which the tobacco manufacturing and sales sector must operate. Through fiscal policies, such as taxation on tobacco

For peer review only - http://bmjopen.bmj.com/site/about/guidelines.xhtml

sales and subsidies to tobacco growers, governments can influence tobacco product production and prices, and, through health promotion and social marketing, they can also influence sociocultural norms. Society, through its traditional, cultural and religious practices, predominantly establishes the cultural norms for any cultural food practices."

Timeline

The protocol for this review is published on PROSPERO (dated July 2nd 2020, Version 2).[30] Searches on the databases mentioned in the protocol were conducted during April-May 2020, following which title and abstract screening for scientific literature was done from July-August 2020. We are planning to conduct the full-text screening in October-November 2020 and hold on the data extraction till the protocol is published.

Ethics and Dissemination Plan

As this study will be a review of published or publicly available data, there are no ethical concerns related to the involvement of humans or any other. Permission for ethics exemption of the review was obtained from the Centre for Chronic Disease Control's Institutional Ethics Committee, India (CCDC_IEC_06_2020; dated 16 April 2020). Data will be collected from publicly available scientific or grey literature through above-mentioned databases. On completion of the review, we will prepare a manuscript for publication in a peer-reviewed journal and present results in national and international conferences.

For peer review only

For peer review only - http://bmjopen.bmj.com/site/about/guidelines.xhtml

Patient and Public Involvement: No patients involved.

Contributors: MA, MM, KS and OD conceptualised the study. MA, AC and NJ drafted the manuscript and MB, SD, SB, SF, RH, ZK, MAR, AR, AV, OD contributed to development of the manuscript as well as finalising the study design. JE provided inputs on data extraction for policy documents, MK reviewed the statistical analysis plan and data extraction plan. RM, KS, AS revised the manuscript critically for intellectual contents. MA and OD supervised the overall process and finalized the manuscript with AC and NJ. All authors approved the final manuscript.

Funding statement: This work is funded by the UK's National Institute for Health Research (NIHR) [ASTRA (Grant Reference Number 17/63/76)]. The views expressed are those of the authors and not necessarily those of the NIHR or the Department of Health and Social Care.

Competing interests: None declared by any author

For peer review only - http://bmjopen.bmj.com/site/about/guidelines.xhtml

Figure 1: Contextual Framework

References

1. World Health Organization. WHO Framework Convention on Tobacco Control. Geneva; 2005.
2. Mehrotra R, Yadav A, Sinha DN, Parascandola M, John RM, Ayo-Yusuf O, et al. Smokeless tobacco control in 180 countries across the globe: call to action for full implementation of WHO FCTC measures. Vol. 20, The Lancet Oncology. Lancet Publishing Group; 2019. p. e208–17.
3. Sinha DN, Suliankatchi RA, Gupta PC, Thamarangsi T, Agarwal N, Parascandola M, et al. Global burden of all-cause and cause-specific mortality due to smokeless tobacco use: Systematic review and meta-analysis. Tob Control. 2018 Jan 1;27(1):35–42.
4. Siddiqi K, Lakshmi Vidyasagaran A, Readshaw A, Croucher R. A Policy Perspective on the Global Use of Smokeless Tobacco. 2017;
5. Siddiqi K, Shah S, Abbas SM, Vidyasagaran A, Jawad M, Dogar O, et al. Global burden of disease due to smokeless tobacco consumption in adults: analysis of data from 113 countries. BMC Med [Internet]. 2015 Dec 17 [cited 2019 Dec 12];13(1):194. Available from: http://bmcmedicine.biomedcentral.com/articles/10.1186/s12916-015-0424-2
6. IARC Working Group on the Evaluation of Carcinogenic Risk to Humans. IARC Monographs on the Evaluation of Carcinogenic Risks to Humans - Smokeless Tobacco and some Tobacco-speicific N-Nitrosamines. Vol. 89, Monograph. Lyon, France: International Agency for Research on Cancer; 2007.
7. Teo KK, Ounpuu S, Hawken S, Pandey M, Valentin V, Hunt D, et al. Tobacco use and risk of myocardial infarction in 52 countries in the INTERHEART study: a case-control study. Lancet. 2006 Aug 19;368(9536):647–58.
8. Niaz K, Maqbool F, Khan F, Bahadar H, Ismail Hassan F, Abdollahi M. Smokeless tobacco (paan and gutkha) consumption, prevalence, and contribution to oral cancer. Vol. 39,

For peer review only - http://bmjopen.bmj.com/site/about/guidelines.xhtml

Epidemiology and health. 2017. p. e2017009.
9. Gupta PC, Subramoney S. Smokeless tobacco use and risk of stillbirth: A cohort study in Mumbai, India. Epidemiology [Internet]. 2006 Jan [cited 2020 Mar 19];17(1):47–51. Available from: http://www.ncbi.nlm.nih.gov/pubmed/16357594
10. Hossain MM, Rahman ME, Khan TH. Maternal smokeless tobacco use and adverse pregnancy outcome. Mymensingh Med J. 2014 Jan 1;23(1):46–51.
11. Suliankatchi RA, Sinha DN. The Human Cost of Tobacco Chewing Among Pregnant Women in India: A Systematic Review and Meta-analysis. J Obstet Gynecol India [Internet]. 2016 Oct 7 [cited 2020 Mar 19];66(S1):161–6. Available from: http://link.springer.com/10.1007/s13224-015-0821-7
12. Levy DT, Mays D, Boyle RG, Tam J, Chaloupka FJ. The Effect of Tobacco Control Policies on US Smokeless Tobacco Use: A Structured Review. Vol. 20, Nicotine & tobacco research : official journal of the Society for Research on Nicotine and Tobacco. 2017. p. 3–11.
13. Tata Institute of Social Sciences, Ministry of Health and Family Welfare Government of India. Global Adult Tobacco Survey GATS 2 India. 2017;
14. Global Adult Tobacco Survey Bangladesh Factsheet [Internet]. 2017. Available from: http://bbs.portal.gov.bd/sites/default/files/files/bbs.portal.gov.bd/page/57def76a_aa3c_46e3_9f80_53732eb94a83/GATS_BAN_2017_Fact Sheet.pdf
15. Alpert HR, Vardavas CI, Chaloupka FJ, Vozikis A, Athanasakis K, Kyriopoulos I, et al. The recent and projected public health and economic benefits of cigarette taxation in Greece. Tob Control [Internet]. 2014 Sep [cited 2020 Mar 19];23(5):452–4. Available from: http://www.ncbi.nlm.nih.gov/pubmed/23467654
16. Siddiqi K, Mishu MP. Smokeless tobacco: Why does it need special attention? Respirology [Internet]. 2019 Aug 13 [cited 2019 Dec 12];24(8):720–1. Available from: https://onlinelibrary.wiley.com/doi/abs/10.1111/resp.13612
17. National Cancer Institute and Centers for Disease Control and Prevention. Smokeless Tobacco and Public Health: A Global Perspective. Bethesda, MD; 2014.
18. Australian Government Department of Health. Smoking and tobacco laws in Australia [Internet]. [cited 2020 Jun 5]. Available from: https://www.health.gov.au/health-topics/smoking-and-tobacco/about-smoking-and-tobacco/smoking-and-tobacco-laws-in-australia
19. Pimple S, Gunjal S, Mishra GA, Pednekar MS, Majmudar P, Shastri SS. Compliance to Gutka ban and other provisons of COTPA in Mumbai. Indian J Cancer. 2014 Oct 1;51(5):S60–6.
20. Siddiqi, K., Husain, S., Vidyasagaran, A. et al. Global burden of disease due to smokeless

For peer review only - http://bmjopen.bmj.com/site/about/guidelines.xhtml

tobacco consumption in adults: an updated analysis of data from 127 countries. BMC Med 18, 222 (2020). https://doi.org/10.1186/s12916-020-01677-9

21. Thomas BH, Ciliska D, Dobbins M, Micucci S. A process for systematically reviewing the literature: Providing the research evidence for public health nursing interventions. Worldviews Evidence-Based Nurs [Internet]. 2004 Sep [cited 2020 Mar 19];1(3):176–84. Available from: http://www.ncbi.nlm.nih.gov/pubmed/17163895

22. Armijo-Olivo S, Stiles CR, Hagen NA, Biondo PD, Cummings GG. Assessment of study quality for systematic reviews: A comparison of the Cochrane Collaboration Risk of Bias Tool and the Effective Public Health Practice Project Quality Assessment Tool: Methodological research. J Eval Clin Pract [Internet]. 2012 Feb [cited 2020 Mar 19];18(1):12–8. Available from: http://www.ncbi.nlm.nih.gov/pubmed/20698919

23. Hoffmann TC, Glasziou PP, Boutron I, Milne R, Perera R, Moher D, et al. Better Reporting of Interventions: Template for Intervention Description and Replication (TIDieR) Checklist and Guide. BMJ [Internet]. 2014 [cited 2020 Jul 9];348. Available from: http://www.bmj.com/content/348/bmj.g1687

24. Higgins JP, Thompson SG, Deeks JJ, Altman DG. Measuring inconsistency in meta-analyses. BMJ 2003;327(7414):557-60.

25. Fleiss J. The statistical basis of meta-analysis. Statistical Methods in Medical Research 1993;2:121-45.

26. Swinburn, B., Sacks, G., Vandevijvere, S., Kumanyika, S., Lobstein, T., Neal, B., Barquera, S., Friel, S., Hawkes, C., Kelly, B. and L'Abbé, M., 2013. INFORMAS (I nternational N etwork for F ood and O besity/non-communicable diseases R esearch, M onitoring and A ction S upport): overview and key principles. Obesity reviews, 14, pp.1-12

27. Bandura, A., (1986). Social Foundations of Thought and Action: A Social Cognitive Theory. Prentice-Hall. P. 24.

28. Messina J, Freeman C, Rees A, Goyder E, Hoy A, Ellis S, Ainsworth N. A systematic review of contextual factors relating to smokeless tobacco use among South Asian users in England. Nicotine & Tobacco Research. 2013 May 1;15(5):875-82.

29. Farhadmollashahi L. Sociocultural reasons for smokeless tobacco use behavior. Int J High Risk Behav Addict. 2014;3(2):e20002. Published 2014 Jun 1. doi:10.5812/ijhrba.20002

30. Arora M, Chugh A, Jain N, Mishu MP, Boeckmann M, Dahanayake S, et al. The global impact of tobacco control policies on smokeless tobacco use: a systematic review. PROSPERO Int Prospect Regist Syst Rev [Internet]. 2020 ; Available from: https://www.crd.york.ac.uk/prospero/display_record.php?RecordID=191946

For peer review only - http://bmjopen.bmj.com/site/about/guidelines.xhtml

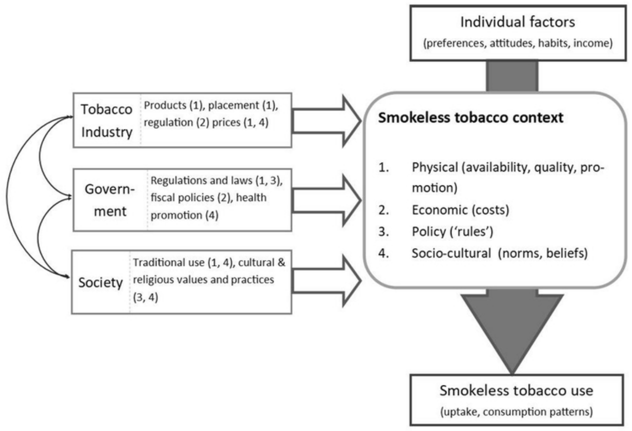

Contextual Framework

100x70mm (300 x 300 DPI)

SUPPLEMENTARY FILE 1_SCIENTIFIC LITERATURE SEARCH STRATEGY

Database: Ovid MEDLINE(R) ALL <1946 to April 14, 2020>

Search Strategy:

----

1 Tobacco, Smokeless/ (3622)

2 (tobacco adj4 (SLT or ST)).ti,ab,kw. (543)

3 (tobacco adj4 (chew$ or dipping or eating or loose-leaf or oral or plug or sneeze or tablet$ or toothpaste or twist$)).ti,ab,kw. (2647)

4 (al-shammah or chemma or chimo or el-shama or ghutkha or gudakhu or gul or gutkha or gutka or iqmik or jarda or khaini or khiwam or kiwan or makla or mawa or mishri or nass or nasvay or naswar or neffa or nufha or paan or quid or sadapata or shammah or snuff or snuif or snus or taaba or tambakoo or tenfeha or tombak or tombol or toombak or tuibur or vizapatta or zarda).ti,bt,ab,kw. (4355)

5 (pan adj4 (tobacco or smokeless)).ti,ab,kw. (75)

6 (nas adj4 (tobacco or smokeless)).ti,ab,kw. (5)

7 1 or 2 or 3 or 4 or 5 or 6 (8157)

8 Public Policy/ or Government Regulation/ or Social Control, Formal/ or Taxation/ (62112)

9 (policies or policy or regulation$ or rule$ or law$ or legislation or tax or taxation or taxes).ti,ab. (1379493)

10 (strateg$ or program$).ti,ab. (1835226)

11 (ban or bans or banning or banned).ti,ab. (14063)

12 Advertising/ or Commerce/ or Marketing/ or Product Packaging/ (44071)

13 (price or prices or pricing or packaging or advertis$ or marketing or sponsor$).ti,ab. (118491)

14 Health Education/ or Health Promotion/ or Mass Media/ (134812)

15 (health warning$ or campaign$ or labelling or label or labels).ti,ab. (213540)

16 (product adj2 regulat$).ti,ab. (1080)

17 (content adj2 disclos$).ti,ab. (65)

18 illicit trade.ti,ab. (118)

19 ((sale$ or selling or sold) adj3 (minor$ or child$)).ti,ab. (390)

20 Smoking Cessation/ (28128)

21 8 or 9 or 10 or 11 or 12 or 13 or 14 or 15 or 16 or 17 or 18 or 19 or 20 (3456116)

22 7 and 21 (2199)

For peer review only

For peer review only - http://bmjopen.bmj.com/site/about/guidelines.xhtml

23 (smokeless tobacco adj3 (cessation or prevent$ or stop$)).ti,ab. (127)

24 22 or 23 (2256)

25 neonatal abstinence syndrome.ti,ab. (916)

26 NASS Patient Satisfaction Index.ti,ab. (5)

27 Normal Astacus saline.ti,ab. (1)

28 naphthenic acid.ti,ab. (136)

29 nicotianamine synthase.ti,ab. (82)

30 25 or 26 or 27 or 28 or 29 (1140)

31 24 not 30 (2253)

32 limit 31 to yr="2005 -Current" (1601)

****

<1>

UI - 32279158

TI - Baseline characteristics of American Indian smokeless tobacco users participating in two pilot cessation studies.

SO - Journal of Community Health. 2020 Apr 11

AU - Lewis CS

AU - Nazir N

AU - Daley SM

AU - Pacheco J

AU - Goeckner RT

AU - Hale JW

AU - Gunville JA

AU - Rahman F

AU - Choi WS

AU - Daley CM

FA - Lewis, Charley S

FA - Nazir, Niaman

FA - Daley, Sean M

For peer review only

For peer review only - http://bmjopen.bmj.com/site/about/guidelines.xhtml

FA - Pacheco, Joseph

FA - Goeckner, Ryan T

FA - Hale, Jason W

FA - Gunville, Jordyn A

FA - Rahman, Fatima

FA - Choi, Won S

FA - Daley, Christine Makosky

PT - Journal Article

For peer review only

For peer review only - http://bmjopen.bmj.com/site/about/guidelines.xhtml

PRISMA-P (Preferred Reporting Items for Systematic review and Meta-Analysis Protocols) 2015 checklist: recommended items to address in a systematic review protocol*

Section and topic	Item No	Checklist item	Status	Corresponding Page number/Line number in the Manuscript
**ADMINISTRATIVE INFORMATION**				
Title:				
Identification	1a	Identify the report as a protocol of a systematic review	✓	Page 3, Line 3
Update	1b	If the protocol is for an update of a previous systematic review, identify as such	Not Applicable	
Registration	2	Mention Registration Number (such as for PROSPERO)	CRD42020191946	Page 2, Line 27
Authors:				
Contact	3a	Provide name, institutional affiliation, e-mail address of all protocol authors; provide physical mailing address of corresponding author	✓	Page 1, Line 4-21
Contributions	3b	Describe contributions of protocol authors and identify the guarantor of the review	✓	
Amendments	4	If the protocol represents an amendment of a previously completed or published protocol, identify as such and list changes; otherwise, state plan for documenting important protocol amendments	Not Applicable	Page 14, Line 2-4
Support:				
Sources	5a	Indicate sources of financial or other support for the review	✓	Page 14, Line 5-7
Sponsor	5b	Provide name for the review funder and/or sponsor	✓	Page 14, Line 5-7
Role of sponsor or funder	5c	Describe roles of funder(s), sponsor(s), and/or institution(s), if any, in developing the protocol	✓	Page 1, Line 16-21; and Page 14, Line 5-7
**INTRODUCTION**				
Rationale	6	Describe the rationale for the review in the context of what is already known	✓	Page 5, Line 11-14
Objectives	7	Provide an explicit statement of the question(s) the review will address with reference to participants, interventions, comparators, and outcomes (PICO)	✓	Page 5, Line 16-24

For peer review only - http://bmjopen.bmj.com/site/about/guidelines.xhtml

**METHODS**				
Eligibility criteria	8	Specify the study characteristics (such as PICO, study design, setting, time frame) and report characteristics (such as years considered, language, publication status) to be used as criteria for eligibility for the review	✓	Page 5, Line 26-34; Page 6 Line 1-32; Page 7, Line 1-33; Page 8, Line 1-16; Page 7, Line 25-27
Information sources	9	Describe all intended information sources (such as electronic databases, contact with study authors, trial registers or other grey literature sources) with planned dates of coverage	✓	Page 7, Line 29-33; Page 8, Line 1-16
Search strategy	10	Present draft of search strategy to be used for at least one electronic database, including planned limits, such that it could be repeated	✓	Page 8, Line 18-34; Page 9, Line 1-25
Study records:				
Data management	11a	Describe the mechanism(s) that will be used to manage records and data throughout the review	✓	Page 9, Line 27-29
Selection process	11b	State the process that will be used for selecting studies (such as two independent reviewers) through each phase of the review (that is, screening, eligibility and inclusion in meta-analysis)	✓	Page 9, Line 31-34
Data collection process	11c	Describe planned method of extracting data from reports (such as piloting forms, done independently, in duplicate), any processes for obtaining and confirming data from investigators	✓	Page 10, Line 1-8
Data items	12	List and define all variables for which data will be sought (such as PICO items, funding sources), any pre-planned data assumptions and simplifications	✓	Page 10, Line 18-32
Outcomes and prioritization	13	List and define all outcomes for which data will be sought, including prioritization of main and additional outcomes, with rationale	✓	Page 7, Line 7-23
Risk of bias in individual studies	14	Describe anticipated methods for assessing risk of bias of individual studies, including whether this will be done at the outcome or study level, or both; state how this information will be used in data synthesis	✓	Page 10, Line 10-15
Data synthesis	15a	Describe criteria under which study data will be quantitatively synthesised	✓	Page 10, Line 17-32; Page 11 Line 1-16
	15b	If data are appropriate for quantitative synthesis, describe planned summary measures, methods of handling data and methods of combining data from studies, including any planned exploration	✓	

For peer review only - http://bmjopen.bmj.com/site/about/guidelines.xhtml

For peer review only

		of consistency (such as I^2, Kendall's τ)		
	15c	Describe any proposed additional analyses (such as sensitivity or subgroup analyses, meta-regression)		Page 11, Line 1-16
	15d	If quantitative synthesis is not appropriate, describe the type of summary planned	✓ (described for narrative synthesis)	Page 10, Line 18-27
Meta-bias(es)	16	Specify any planned assessment of meta-bias(es) (such as publication bias across studies, selective reporting within studies)	✓	Page 11, Line 1-8
Confidence in cumulative evidence	17	Describe how the strength of the body of evidence will be assessed (such as GRADE)	✓	Page 10, Line 17-32; Page 11 Line 1-16

*** It is strongly recommended that this checklist be read in conjunction with the PRISMA-P Explanation and Elaboration (cite when available) for important clarification on the items. Amendments to a review protocol should be tracked and dated. The copyright for PRISMA-P (including checklist) is held by the PRISMA-P Group and is distributed under a Creative Commons Attribution Licence 4.0.**

From: Shamseer L, Moher D, Clarke M, Ghersi D, Liberati A, Petticrew M, Shekelle P, Stewart L, PRISMA-P Group. Preferred reporting items for systematic review and meta-analysis protocols (PRISMA-P) 2015: elaboration and explanation. BMJ. 2015 Jan 2;349(jan02 1):g7647.

For peer review only

Addressing Smokeless Tobacco and Building Capacity in South Asia (ASTRA) – Policy Workstream

Data Extraction Form

SECTION I: General Information and Identification

1.	Title of the Article/ Document	
2.	Study ID *(surname of first author and year study was published e.g. Smith 2001)*	
3.	Report ID *(for projects or studies with multiple report - if different to Study ID e.g. Smith 2001_01)*	
4.	Report IDs of other reports of this study *(e.g. duplicate publications, follow-up studies)*	
5.	Date form completed (dd/mm/yyyy)	
6.	Initials of person extracting data	
7.	Full reference with URL	
8.	Type of Document	☐Scientific article<br>☐Government Report<br>☐Policy Document<br>☐Non-government report<br>☐Commentary<br>☐Editorial<br>☐Government Circular<br>☐Others
9.	Study author contact details	
10.	Source of document<br>*(If ministry website, mention which ministry – Health, Environment, Commerce etc.)*	☐Academic journal<br>☐Ministry website, name:<br>____

		☐Google<br>☐Other, name:____
11.	Country (in which study was conducted/policy document is based)	
12.	Duration of study *(start and end date)*	
13.	State funding source	
14.	Ethics approval obtained for the study (Y/N)	

SECTION II: DETAILS OF SMOKELESS TOBACCO POLICY (specific to RQ1)

1.	Population (study participants)	☐Smokers<br>☐Smokeless Tobacco Users<br>☐Dual Users<br>☐Any other, please specify:
2.	Age group of study participants (adolescents/young adults/adults)	☐ All age groups<br>☐adults, age range: _______________________<br>☐children/youth, age range: _______________________<br>☐other, age range: _______________________<br>☐comment/warning message (if any):<br>__________________________
3.	Gender distribution of participants	Total number of males (%):<br>Total number of females (%):
4.	Setting of the population ?(national or sub-national)	
5.	Number of participants/sample size	

For peer review only

	**INTERVENTION (POLICY) DESCRIPTION**	
	**INTERVENTION (POLICY) 1 (replicate the entire section in case of more than 1 policy)**	
1.	Intervention (policy) focus	**FCTC Policies**<br>☐Pricing and taxation (Article 6)<br>☐Product regulation (Article 9 and 10)<br>☐Packaging and health warnings (Article 11)<br>☐Education, communication, training, and public awareness (Article 12)<br>☐Advertisement, promotion and sponsorship bans (Article 13)<br>☐Cessation (Article 14)<br>☐Illicit trade (Article 15)<br>☐Sales to and by minors (Article 16)<br>**Non-FCTC Policies**<br>☐Complete ban<br>☐Partial ban<br>☐Import ban<br>☐Other policies mentioned to control ST (agriculture, environment etc.), please specify____<br>_______________
2.	Comparator *(usual care/control etc.)*	☐Reported/Describe:<br>____<br>☐Not reported (but should be reported)<br>☐Not applicable
Description of intervention (using TIDieR checklist: https://www.equator-network.org/reporting-guidelines/tidier/)		
3.	Brief name<br>(name or phrase that describes the policy in the document)	☐Present/Describe:<br>____<br>☐Absent (but should be reported)

For peer review only - http://bmjopen.bmj.com/site/about/guidelines.xhtml

		☐Not applicable (when it is legitimately not relevant)
4.	Why?<br>(Describe any rationale, theory, or goal of the elements essential to the policy)	☐Present/Describe:<br>____<br>☐Absent (but should be reported)<br>☐Not applicable (when it is legitimately not relevant)
5.	What materials<br>(any physical or informational materials used for the policy)	☐Present/Describe:<br>____<br>☐Absent (but should be reported)<br>☐Not applicable (when it is legitimately not relevant)
6.	What procedures<br>(procedures, activities, and/or processes used in the policy)	☐Present/Describe:<br>____<br>☐Absent (but should be reported)<br>☐Not applicable (when it is legitimately not relevant)
7.	Who provided<br>(For each category of intervention provider (e.g. psychologist, nursing assistant), describe their expertise, background and any specific training given; N/A for non-human provider modes)	☐Present/Describe:<br>____<br>☐Absent (but should be reported)<br>☐Not applicable (when it is legitimately not relevant)
8.	How<br>(modes of delivery (e.g. face-to-face or by some other mechanism, such as internet or telephone) of the intervention and whether it was provided individually or in a group)	☐Present/Describe:<br>____<br>☐Absent (but should be reported)<br>☐Not applicable (when it is legitimately not relevant)
9.	Where<br>(Describe the type(s) of location(s) where the policy occurred, including any necessary infrastructure or relevant features)	☐Present/Describe:<br>____<br>☐Absent (but should be reported)<br>☐Not applicable (when it is legitimately not relevant)
10.	When and how much<br>(period of time covered by the policy and any specification on frequency and intensity)	☐Present/Describe:<br>____<br>☐Absent (but should be reported)<br>☐Not applicable (when it is legitimately not relevant)

For peer review only

11.	Tailoring<br><br>(If it was planned to be personalised, titrated or adapted for a specific population, then describe what, why, when, and how)	☐Present/Describe: ______________________________<br>☐Absent (but should be reported)<br>☐Not applicable (when it is legitimately not relevant)
12.	Modifications<br><br>(any modified made to the policy during the course of the study, describe the changes (what, why, when, and how))	☐Present/Describe: ______________________________<br>☐Absent (but should be reported)<br>☐Not applicable (when it is legitimately not relevant)
13.	How well implemented (plan)<br><br>(whether policy adherence was assessed, and if any strategies were used to maintain and improve adherence)	☐Present/Describe: ______________________________<br>☐Absent (but should be reported)<br>☐Not applicable (when it is legitimately not relevant)
14.	How well implemented (actual)<br><br>(if policy adherence was assessed, describe the extent to which it was implemented as planned)	☐Present/Describe: ______________________________<br>☐Absent (but should be reported)<br>☐Not applicable (when it is legitimately not relevant)
	Contextual specification of intervention	
15.	Is the extent of policy enforcement described in the document?	☐Yes, National/federal level<br>☐Yes, Regional/state-level/provincial<br>☐No<br><br>If Yes, Describe the extent:________________
16.	Enforcers/regulators of the policy (Government body enforcing or regulating the policy)	☐Ministry/Department/Division of Health<br>☐Ministry/Department/Division of Commerce<br>☐Ministry/Department/Division of Finance<br>☐Ministry/Department/Division of Environment<br>☐Food and Drug Administration<br>☐ Others, please specify____

For peer review only

17.	Does this document identify if stakeholders were involved in developing/modifying the policy?	☐Yes<br>☐No
18.	If Yes in Point 5, select all the stakeholders that were involved in developing/modifying the policy	☐Federal Government/National Government<br>☐Provincial/State/Regional Government<br>☐Health Care Organisations<br>☐Experts<br>☐Regulators<br>☐Professional Organisations (non-regulatory)<br>☐Clinicians<br>☐Patients<br>☐Researchers<br>☐Others (specify)____________________________<br>☐Not available
19.	Does the document describe any policy drivers, e.g., preamble or rationale for introducing policies (like media coverage, political will, public health concern etc.)?	☐Yes<br>☐No<br><br>if yes, specify details:
20.	Does the document evaluate or mention evaluation of the policy's effectiveness?	☐Evaluates impact *(complete Section III)*<br>☐Mentions evaluation of impact *(in methods)*<br>*State/provide reference of the article/document with details of the impact evaluation:*<br>☐None of the above
21.	Any other details (limitations or other observations)	

SECTION III: DETAILS OF IMPACT OF SMOKELESS TOBACCO POLICIES (specific to RQ2)

A. Methods

			Location in text (Page #/ Figure/Table)
1.	Study objectives *(as stated in the study)*		
2.	Design	☐Randomized controlled trial<br>☐Controlled clinical trial<br>☐Cohort analytic (two groups pre+post)<br>☐Case-control<br>Cross sectional (surveys)<br>☐Cohort (one group pre+post (before and after))<br>☐Interrupted time series<br>☐Other specify_______________________________<br>☐Not specified	
3.	Sampling technique with details	☐Random sampling<br>☐Purposive sampling<br>☐Snowball sampling<br>☐Cluster sampling<br>☐Any other, please specify:<br>_______________________________<br>☐Not specified	

For peer review only

4.	Is the analysis of the study conducted at individual level?	☐No<br>☐Yes, please give details:_________	
5.	Is the analysis of the study conducted at group level?	☐No<br>☐Yes, please give details:___________	

B. Outcomes

			Location in text (Page #/ Figure/Table)
	**PRIMARY OUTCOME 1 (replicate the section in case of more than one PRIMARY outcome)**		
1.	Outcome name (e.g. quit rate)		
2.	Outcome definition		
3.	Time points measured		
4.	Time since policy implementation		
5.	Time points reported		

For peer review only

6.	Total N (% - *at this stage of follow-up as % of N at time of enrolment in study)*		
7.	N (%) with outcome		
8.	Effect estimate *(e.g. Odds Ratio/Prevalence percentage/risk ratio/mean/median)*		
9.	Unit of effect estimate *(e.g. O*dds Ratio, percentage, mean etc.*)*		
10	Confidence/precision intervals of effect estimate *(e.g. 95% CI, IQR, SD, SR etc.)*		
11	Is tool validated for population of interest		
	**INTERMEDIATE OUTCOME**		
	**Details**		
	**UNINTENDED OUTCOME**		
	**Details**		

For peer review only

	Notes:

C. COMPARATORS

			Location in text (Page #/ Figure/Table)
	**COMPARATOR 1 (Replicate the section in case of more than one comparator)**		
1.	Comparator Name (e.g. quit rate)		
2.	Comparator definition		
3.	Time points measured		
4.	Time since policy implementation		
5.	Time points reported		
6.	Total N (% - *at this stage of follow-up as % of N at time of enrolment in study)*		
7.	N (%) with outcome		
8.	Effect estimate *(e.g. Odds Ratio/Prevalence percentage/risk ratio/mean/median)*		

For peer review only

9.	Unit of effect estimate *(e.g. O*dds Ratio, percentage, mean etc.*)*		
10.	Confidence/precision intervals of effect estimate *(e.g. 95% CI, IQR, SD, SR etc.)*		

D. Limitation and Mitigation Strategy (author identified)

			Location in text (Page #/ Figure/Table)
1.	Strength		
2.	Limitation		

E. Conclusions

			Location in text (Page #/ Figure/Table)
1.	Key Conclusion of Study Author/s		

For peer review only

F. Risk of bias (quality assessment)

1. **SELECTION BIAS**	
a. Are the individuals selected to participate in the study likely to be representative of the target population?	☐Very likely<br>☐Somewhat likely<br>☐Not likely<br>☐Can't tell
b. What percentage of selected individuals agreed to participate?	☐80-100% agreement<br>☐60-79% agreement<br>☐Less than 60% agreement<br>☐Not applicable<br>☐Can't tell
Rate this section (selection bias)	☐1 Strong<br>☐2 Moderate<br>☐3 Weak
2. **STUDY DESIGN**	
a. Indicate the study design	☐Randomized controlled trial<br>☐Controlled clinical trial<br>☐Cohort analytic (two groups pre+post)<br>☐Case-control<br>Cross sectional (surveys)<br>☐Cohort (one group pre+post (before and after))<br>☐Interrupted time series<br>☐Other specify________________________________<br>☐Can't tell
b. Was the study described as randomized? (If No, go to component 3)	☐No<br>☐Yes

For peer review only

c. If yes, was the method of randomization described?	☐No<br>☐Yes
d. If yes, was the method appropriate?	☐No<br>☐Yes
Rate this section (study design)	☐1 Strong<br>☐2 Moderate<br>☐3 Weak
3. **CONFOUNDERS**	
a. Were there important differences between groups prior to intervention?	☐Yes<br>☐No<br>☐Can't tell
The following are examples of confounders	☐Race<br>☐Sex<br>☐Marital status/family<br>☐Age<br>☐SES (income or class)<br>☐Education<br>☐Health status<br>☐Pre-intervention score on outcome measure
b. If yes, indicate the percentage of relevant confounders that were controlled (either in the design (e.g., stratification, matching) or analysis)?	☐80-100% (most)<br>☐60-79% (some)<br>☐Less than 60% (few or none)<br>☐Can't tell
Rate this section	☐1 Strong<br>☐2 Moderate<br>☐3 Weak
**4. BLINDING**	
a. Was (were) the outcome assessor(s) aware of the intervention or exposure status of participants?	☐Yes<br>☐No<br>☐Can't tell

For peer review only

b. Were the study participants aware of the research question?	☐Yes<br>☐No<br>☐Can't tell
Rate this section	☐1 Strong<br>☐2 Moderate<br>☐3 Weak
**5. DATA COLLECTION METHODS**	
a. Were data collection tools shown to be valid?	☐Yes<br>☐No<br>☐Can't tell
b. Were data collection tools shown to be reliable?	☐Yes<br>☐No<br>☐Can't tell
Rate this section	☐1 Strong<br>☐2 Moderate<br>☐3 Weak
**6. WITHDRAWALS AND DROP-OUTS**	
a. Were withdrawals and drop-outs reported in terms of numbers and/or reasons per group	☐Yes<br>☐No<br>☐Can't tell<br>☐Not applicable (i.e. one time surveys or interviews)
b. Indicate the percentage of participants completing the study (if the percentage differs by groups, record the lowest)	☐80-100%<br>☐60-79%<br>☐Less than 60%<br>☐Can't tell
Rate this section	☐1 Strong<br>☐2 Moderate<br>☐3 Weak
**7. INTERVENTION INTEGRITY**	

For peer review only

a. What percentage of participants received the allocated intervention or exposure of interest?	☐80-100%<br>☐60-79%<br>☐Less than 60%<br>☐Can't tell
b. Was the consistency of the intervention measured?	☐Yes<br>☐No<br>☐Can't tell
c. Is it likely that subjects received an unintended intervention (contamination or co-intervention) that may influence the results	☐Yes<br>☐No<br>☐Can't tell
**8. ANALYSES**	
a. Indicate the unit of allocation (select one)	☐Community<br>☐Organisation/institution<br>☐Practice/office<br>☐Individual
b. Indicate the unit of analysis (select one)	☐Community<br>☐Organisation/institution<br>☐Practice/office<br>☐Individual
c. Are the statistical methods appropriate for the study design?	☐Yes<br>☐No<br>☐Can't tell
d. Is the analysis performed by intervention allocation status (i.e. intention to treat) rather than the actual intervention received?	☐Yes<br>☐No<br>☐Can't tell
**COMPONENT RATINGS**	
a. Selection Bias	☐1 Strong<br>☐2 Moderate<br>☐3 Weak
b. Study Design	☐1 Strong

For peer review only

	☐2 Moderate<br>☐3 Weak
1 Confounders	☐1 Strong<br>☐2 Moderate<br>☐3 Weak
2 Blinding	☐1 Strong<br>☐2 Moderate<br>☐3 Weak
3 Data collection method	☐1 Strong<br>☐2 Moderate<br>☐3 Weak
4 Withdrawals and drop-outs	☐1 Strong<br>☐2 Moderate<br>☐3 Weak
**GLOBAL RATING FOR THIS PAPER (SELECT ONE)**	☐1 Strong<br>☐2 Moderate<br>☐3 Weak
(With both reviewers discussing the ratings)<br>Is there a discrepancy between the reviewers with respect to the component (a. – f.)	☐No<br>☐Yes
If yes, indicate the reason for discrepancy	☐Oversight<br>☐Differences in interpretation of criteria<br>☐Differences in interpretation of study
**Final Decision of both reviewers (select one)**	☐1 Strong<br>☐2 Moderate<br>☐3 Weak

For peer review only
